# Consumers Modulate Effects of Plant Diversity on Community Stability

**DOI:** 10.1111/ele.70103

**Published:** 2025-03-20

**Authors:** Maowei Liang, Seraina L. Cappelli, Elizabeth T. Borer, David Tilman, Eric W. Seabloom

**Affiliations:** ^1^ Cedar Creek Ecosystem Science Reserve University of Minnesota East Bethel Minnesota USA; ^2^ Department of Ecology, Evolution, and Behavior University of Minnesota St. Paul Minnesota USA; ^3^ Bren School of Environmental Science and Management University of California Santa Barbara California USA

**Keywords:** asynchrony, biodiversity, complexity, herbivory, insurance effects, stability, trophic interactions

## Abstract

Biotic complexity, encompassing both competitive interactions within trophic levels and consumptive interactions among trophic levels, plays a fundamental role in maintaining ecosystem stability. While theory and experiments have established that plant diversity enhances ecosystem stability, the role of consumers in the diversity–stability relationships remains elusive. In a decade‐long grassland biodiversity experiment, we investigated how heterotrophic consumers (e.g., insects and fungi) interact with plant diversity to affect the temporal stability of plant community biomass. Plant diversity loss reduces community stability due to increased synchronisation among species but enhances the population‐level stability of the remaining plant species. Reducing trophic complexity via pesticide treatments does not directly affect either community‐ or population‐level stability but further amplifies plant species synchronisation. Our findings demonstrate that the loss of arthropod or fungal consumers can destabilise plant communities by exacerbating synchronisation, underscoring the crucial role of trophic complexity in maintaining ecological stability.

## Graphical abstract

Trophic complexity mediates the direct and indirect effects of plant diversity on community stability. Increasing plant species richness enhances species asynchrony both directly and indirectly (by increasing the effective number of species), thereby promoting community stability. However, the presence of heterotrophic consumers shifts the balance toward indirect pathway effects through trade‐offs.

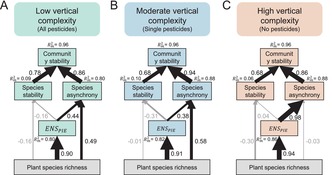

## 1 Introduction

Elton (1958) proposed that ecosystems with greater diversity are inherently more stable against perturbations, attributing this stability to complex food webs that support myriad interactions among species. These interactions occur both within trophic levels (e.g., competitive interactions) and among trophic levels (e.g., consumptive interactions). Since then, the diversity–stability relationship has continued to intrigue ecologists (May 1972; McNaughton 1977; Pimm 1984), with a particular focus on the role of plant diversity (i.e., competitive interactions) (McCann 2000; Hooper et al. 2005; Cardinale et al. 2012; Tilman et al. 2014; Loreau et al. 2021). Many experiments (Tilman et al. 2006; Hector et al. 2010; Schnabel et al. 2021; Wagg et al. 2022), manipulating the number of plant species (e.g., competitive interactions), have supported the theoretical prediction that greater plant diversity enhances the temporal stability of plant community biomass (i.e., plant community stability) (Doak et al. 1998; Tilman et al. 1998; Yachi and Loreau 1999; Lehman and Tilman 2000; Loreau et al. 2021). While those findings confirm that plant diversity stabilises biomass production and buffers ecosystems against interannual environmental variability, the role of consumptive interactions among trophic levels (i.e., trophic interactions) remains poorly understood (McCann 2000; Worm and Duffy 2003; Tilman et al. 2014; Eisenhauer et al. 2024).

Although an increasing number of studies have investigated the effects of trophic interactions on plant community stability (Ives et al. 1999; Ives et al. 2000; Halpern et al. 2005; Thébault and Loreau 2005; Kohli et al. 2019; Zhao et al. 2019; Firkowski et al. 2021; Eschenbrenner and Thébault 2023; Srednick and Swearer 2024), the results have largely been idiosyncratic. Some theoretical models predicted that trophic interactions have a minimal effect on plant community stability (Ives et al. 1999; Ives et al. 2000; Eschenbrenner and Thébault 2023). In contrast, other models suggest that plant–herbivore interactions exert a destabilising effect on plant communities (Thébault and Loreau 2005), with even greater destabilisation when consumer diversity is greater (Zhao et al. 2019) or for multitrophic‐level systems (Firkowski et al. 2021). Some empirical studies have shown that predators (Halpern et al. 2005) or consumers (Kohli et al. 2019) do not significantly alter plant community stability. Other studies in both aquatic (e.g., *Daphnia* (Rakowski and Cardinale 2016)) and terrestrial (e.g., *Ovis* (Liang et al. 2021; Zuo et al. 2023)) systems have found that herbivores can destabilise the plant community. Moreover, in systems in which only single trophic levels are considered, plant diversity stabilises communities primarily through ‘*portfolio*’ or ‘*insurance*’ effects, driven by species asynchrony (i.e., variance among species over time) (Loreau and de Mazancourt 2008; Hector et al. 2010; Hautier et al. 2014; Craven et al. 2018; Schnabel et al. 2021; Liang et al. 2022). However, meta‐analyses suggest that in systems where herbivory is also considered, plant diversity stabilises communities through positive relationships with species stability (i.e., averaging stability at the population level) rather than species asynchrony (Jiang and Pu 2009; Lamy et al. 2020; Xu et al. 2021; Liang et al. 2024; Srednick and Swearer 2024).

**FIGURE 1:**
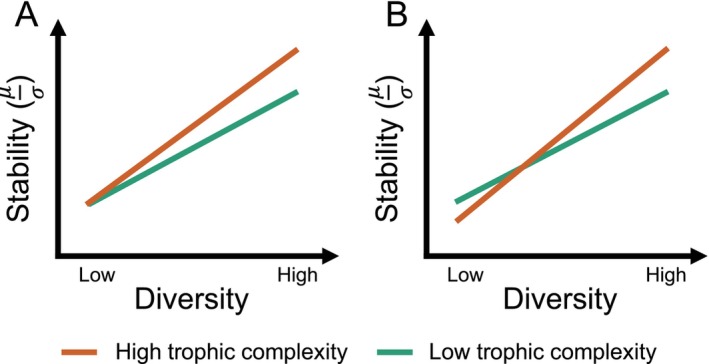
Hypothetical changes in consumers' modulation of the diversity−stability relationships in plant communities. We propose that consumers (e.g., herbivores, pathogens) influence the diversity–stability relationship in plant communities through cascading top‐down effects (e.g., foraging behaviour and pressures, apparent competition, trade‐offs among growth, herbivory defence mechanisms, nutrient acquisition). In high‐diversity plant communities, selective foraging by consumers can reduce the dominance of favourable plant species, allowing other species to persist or increase, potentially enhancing plant community stability. Higher trophic complexity, for example, the presence of diverse consumers, may intensify foraging pressures to consume more targeted species biomass, promoting species coexistence, and further increasing plant community stability (Figure 1A,B). Conversely, in low‐diversity communities, such as monocultures, synchronised biomass fluctuations often result in lower stability than in high‐diversity communities. If plant species in these systems are not preferred by consumers, higher trophic complexity may have negligible effects on stability (Figure 1A). However, when plant species are targeted by consumers, increased trophic complexity may exacerbate foraging pressure, leading to catastrophic biomass loss and reduced stability, as alternative species are unavailable to buffer these impacts (Figure 1B).

Trophic interactions can influence plants through various top‐down processes, which likely vary based on the diversity of host communities, particularly through the foraging behaviour and pressures (Worm and Duffy 2003; Duffy et al. 2007). In diverse plant communities, consumers (e.g., herbivores, pathogens) may stabilise biomass over time (i.e., consumer‐stabilisation hypothesis), likely driven by consumer‐mediated negative conspecific density dependence. In general, the dominant plant species are likely to be foraged by specialised consumers, allowing other species to persist or increase (Milchunas et al. 1988; Olff and Ritchie 1998). This can maintain or even increase plant community stability, particularly in heterogeneous environments (Zuo et al. 2023; Trepel et al. 2024). Higher trophic complexity (e.g., the presence of diverse consumers) will likely amplify the stabilising effect of consumers via dietary specialisation, which may promote species coexistence and further enhance plant community stability (Figure 1A,B). In contrast, species‐poor plant communities (e.g., monocultures) generally are less stable than diverse communities due to synchronised biomass fluctuations over time (Loreau and de Mazancourt 2008). Increasing trophic complexity in these systems may leave plant community stability unchanged if herbivores or pathogens exert minimal pressure on plant species (Figure 1A). However, if specialised consumers target individual plant species, increased trophic complexity can intensify foraging pressure, reducing stability, as no other species are present to compensate (Figure 1B). Thus, whether observed stability attributed to plant diversity is mediated by consumers and how consumers influence the diversity–stability relationship remain open questions.

Despite the potential for interplay between trophic complexity and plant diversity to impact plant community stability, it has rarely been tested empirically. Most experimental tests of the diversity–stability relationships in plant communities have been conducted in the presence of diverse consumers, such as mutualists, pathogens and herbivores of many types (Mulder et al. 1999; Ebeling et al. 2008; Borer et al. 2015; Li et al. 2023). However, analyses of these studies have primarily focused on mechanisms related to the effects of plant diversity (McCann 2000; Worm and Duffy 2003; Tilman et al. 2014; Loreau et al. 2021; Eisenhauer et al. 2024). This disconnection underscores a critical gap: the potential role of consumers in destabilising/stabilising plant communities. Here, we used a long‐term field experiment established in 2008, in which we manipulated the presence of different heterotrophic consumer groups (i.e., trophic complexity) (Borer et al. 2015; Seabloom et al. 2017; Seabloom et al. 2018; Kohli et al. 2019; Zaret et al. 2022). The consumer manipulation experiment is nested within the longest‐running plant diversity experiment, initiated in 1994 (Tilman et al. 1997). To experimentally determine how plant diversity and trophic complexity jointly impact plant community stability, we created three different trophic communities of low (three heterotrophic consumer groups excluded: insects, foliar fungi AND soil oomycetes), moderate (one heterotrophic consumer group excluded: insects, foliar fungi OR soil oomycetes) and high trophic complexity (no heterotrophic consumer groups excluded) in subplots of plant monocultures, four species and 16 species mixtures (Borer et al. 2015). We tested mechanistic hypotheses using 15 years of annual plant biomass data (2009–2023). Specifically, we hypothesised that greater trophic complexity (i.e., the presence of diverse consumers) would stabilise temporal biomass fluctuations in plant communities by increasing species asynchrony compared to plant communities with low trophic complexity. We tested the generality of this hypothesis by manipulating different trophic groups. Thus, we expected that increasing trophic complexity would consistently amplify the stabilising influence of plant diversity.

## 2 Material and Methods

### 2.1 Study Area

We carried out this research at the Cedar Creek Ecosystem Science Reserve, located in East Bethel, in central Minnesota, USA (45.4° N and 93.2° W). The region is characterised by a humid continental climate with warm summers and cold winters. Average annual precipitation in the area is approximately 750 mm, with summer months usually receiving the most rainfall. The mean annual temperature is 6°C, with winter temperatures often remaining below freezing, and summers generally warm (Reich et al. 2018). The study site lies on glacial sand, and the sandy soils are very nutrient‐poor and have low productivity relative to other grasslands (Fay et al. 2015).

### 2.2 Plant Diversity Experiment

In 1994, we established 168 plots, each 9 × 9 m, where we manipulated plant species richness. Each plot was randomly assigned to a species diversity treatment (1, 2, 4, 8 or 16 species), with the composition of each randomly drawn from a pool of 18 native perennial species (Tilman et al. 1997; Tilman et al. 2006). The plant species pool comprised four species each of C4 grasses, C3 grasses, legumes and forbs, in addition to two woody species. The plot richness and composition treatments have been maintained with annual weeding since the initiation of the experiment. To eliminate potential impacts of deer and small mammals, we fenced the entire experiment. Plots with 16 plant species represent high complexity with extensive competitive interactions. Conversely, the plant monoculture plots represent simpler systems with minimal competitive interactions. These plots provide a gradient of competitive complexity, allowing us to study stability across a gradient of complexity but within a trophic level.

### 2.3 Heterotroph Reduction: A Dimension of Trophic Complexity

The field was fenced to exclude mammalian herbivores, thus allowing isolation of the impacts of smaller but ecologically important consumer groups: insect herbivores and fungal pathogens. In 2008, we introduced food‐web manipulations within 33 of the 9 × 9 m diversity plots, including monoculture (1 species, *n* = 15), 4 species (*n* = 9) or 16 species (*n* = 9) treatments. Within each plot, we demarcated five subplots of 1.5 × 2 m and randomly assigned one of five food‐web manipulation treatments to each subplot: Control, Insecticide, Foliar Fungicide, Soil Fungicide and a Combination Treatment of all three pesticides. Pesticides were applied biweekly throughout the growing season, from mid‐April until the end of August each year. In previous studies, we assessed the effects of pesticides by collecting plant leaves from treated subplots to evaluate herbivory damage and foliar chemistry traits. We sampled two focal C4 grasses ( *Andropogon gerardii* and *Schizachyrium scoparium* ) and two focal legumes ( *Lespedeza capitata* and *Lupinus perennis* ). In total, we collected 498 individual plants from the treated subplots. Our findings revealed that insecticide and foliar fungicides reduced the prevalence of insect and fungal pathogen damage, respectively, and also increased total plant biomass (Borer et al. 2015; Seabloom et al. 2017). Additionally, soil fungicide impacted plant community composition, increasing forb abundance (Borer et al. 2015). Note that while our study assesses the effects of trophic complexity treatments through plant responses, we did not directly measure changes in the targeted trophic groups themselves. This limitation should be considered when interpreting our findings. Additional details about the experiment are provided in previous works (Borer et al. 2015; Seabloom et al. 2017; Seabloom et al. 2018; Kohli et al. 2019; Zaret et al. 2022). In this study, the Control (without any pesticides) represents the high complexity condition with the full community intact and extensive interspecific interactions among consumer groups and their host plants. Applying all pesticides (Insecticide, Foliar Fungicide, Soil Fungicide) creates a low complexity community, and the use of individual pesticides creates communities of moderate complexity.

### 2.4 Diversity and Stability Calculations

All diversity and stability indices calculated are based on plant biomass. Annually, from 2009 to 2023, we collected aboveground biomass at the peak of the growing season, typically in early August, from each subplot within the experiment. In each plot, we used a handheld clipper to cut all plants rooted in a 6.0×0.1 m strip as close to the ground as possible. The collected plant material was sorted to species, dried to a constant mass and weighed to the nearest 0.001 g. We quantified the Effective Number of Species based on the Probability of Interspecific Encounter ${\mathrm{ENS}}_{\mathrm{PIE}}$, which is equivalent to the inverse of the Simpson index, calculated as $\frac{1}{\sum_{i=1}^{S}p_{i}^{2}}$, where S is the total number of species and $p_{i}$ is the proportion of the community biomass resented by species i. In a mathematical sense, ${\mathrm{ENS}}_{\mathrm{PIE}}$ can be deconstructed into the product of species richness (S) and evenness; thus, ${\mathrm{ENS}}_{\mathrm{PIE}}=\text{plant richness}\times \text{plant evenness}$ (Seabloom et al. 2020). For additional analysis, we also calculated the Shannon–Weiner Diversity indices ${Η}^{\prime }=-\sum_{i=1}^{S}p_{i}\ln\left(p_{i}\right)$, as a second measure of ‘effective’ plant diversity (i.e., $e^{H^{\prime }}$).

Furthermore, we quantified plant community stability using the biomass data over the 15 years. We defined stability by temporal invariability, that is the ratio of mean to standard deviation over time (Tilman et al. 2006). We defined community stability as the stability of the whole plant community, calculated as $\text{community stability}=\frac{\sum_{k}\mu_{\mathrm{ik}}}{\sqrt{\sum_{k,l}v_{\mathrm{ikl}}}}$, where $\mu_{\mathrm{ik}}$ and $v_{\mathrm{ik}}$ denote the temporal mean and variance of the biomass of species k in community i, and $\nu_{\mathrm{ikl}}$ denotes the covariance between species k and species l in community i (Wang et al. 2019). We defined species stability as the weighted average of local species stability among species of the community i, $\text{species stability}=\frac{\sum_{k}\mu_{\mathrm{ik}}}{\sum_{k}\sqrt{v_{\mathrm{ikk}}}}$. Species asynchrony, which represents the variance–covariance matrix, was calculated as $\text{species asynchrony}=\frac{\sum_{k}\sqrt{v_{\mathrm{ikk}}}}{\sqrt{\sum_{k,l}v_{\mathrm{ikl}}}}$. Species asynchrony can be calculated as the ratio of community stability to species stability, that is, species asynchrony=community stability/species stability (Loreau and de Mazancourt 2008; Thibaut and Connolly 2013; Wang et al. 2019). Alternatively, community stability=species asynchrony×species stability. If the variation explained by species asynchrony exceeds that explained by species stability, it suggests that species asynchrony plays a particularly strong role in driving community stability, and vice versa.

Because plots are weeded to maintain only planted species, we calculated diversity and stability indices using the biomass of planted species only. Note that both Simpson, Shannon ${Η}^{\prime }$, plant evenness, and species asynchrony equal to 1 in the monoculture treatment due to the mathematical relationships between richness, evenness and diversity metrics. For instance, increasing species richness inherently reduces evenness when S>1. To clarify, we used ‘plant species richness’ to refer to the manipulated richness in our biodiversity experiment, and ‘realised plant diversity’ to denote the diversity calculated as ${\mathrm{ENS}}_{\mathrm{PIE}}$ for each treatment, which includes metrics such as the inverse Simpson index and the Shannon H′ index.

### 2.5 Statistical Analysis

To assess the effects of both plant species richness (number of plant species) and trophic complexity (number of heterotrophic groups) on plant biomass and realised plant diversity (${\mathrm{ENS}}_{\mathrm{PIE}}$, plant evenness and Shannon H′ index), we employed linear mixed‐effect models (MEMs) using the R package ‘*nlme*’ (Pinheiro et al. 2025). We analysed annual plant biomass and the diversity indices in response to plant species richness and trophic complexity and their interaction. Plant species richness (1, 4 and 16 species) was included as a continuous variable, trophic complexity (low, moderate, high) as a discrete variable. To account for the experimental design, we included random intercepts per experimental plot (‘*plotID*’) and specific heterotroph reduction treatment (insecticide, foliar fungicide and soil drench fungicide) in the models. We also include ‘*year*’ (continuous variable) as additional fixed effect with an autocorrelation structure corAR1(form = ~ year | plotID). For example: ${\mathrm{vi}}_{\text{annual}}$ ~ plant species richness × trophic complexity × year + random (~1|plotID), correlation = corAR1(form = ~ year | plotID). Additionally, we applied MEMs, for example, ${\mathrm{vi}}_{\mathrm{sta}}$ ~ plant species richness × trophic complexity + random (~1|plotID), to analyse community stability and its components temporal mean and standard deviation of plant biomass in response to plant species richness and trophic complexity and their interaction. Another two components of the mathematical partitioning of community stability, species stability and species asynchrony, were also analysed using the same model structure.

Moreover, to examine the effects of trophic complexity on the determinants of community stability variation, we utilised ordinary least squares linear models (LMs) to analyse bivariate relationships. These LMs predict community stability (response variable) with its constituents (temporal mean and standard deviation of plant biomass, species stability and species asynchrony), as well as diversity metrics (means of the inverse of the Simpson index and Shannon H′ index) across three levels of trophic complexity. Similarly, we applied the same model framework to explore bivariate relationships between these constituents and diversity metrics. We visually checked that all the models meet the assumptions of normality and independence of errors.

Finally, to investigate the direct and indirect effects of plant species richness on plant community stability, we used structural equation models (SEMs) across three distinct levels of trophic complexity. Initially, we developed a null SEM, outlining all plausible pathways informed by prior research (Figure S1). This foundational model was refined into a saturated model, allowing for the comparison of pathway coefficients across different treatments. The null SEM captured the direct effects of plant species richness on realised plant diversity (*Plant species richness → Realised plant diversity*), species stability (*Plant species richness → Species stability*), and species asynchrony (*Plant species richness → Species asynchrony*). Additionally, the SEMs incorporated the indirect effects of plant species richness on species stability (*Plant species richness → Realised plant diversity → Species stability*) and species asynchrony (*Plant species richness → Realised plant diversity → Species asynchrony*). Ultimately, these comparative SEMs enabled us to discern the differing pathway effects of plant species richness on community stability across trophic complexity levels. We ran the SEMs using both the inverse of the Simpson index and Shannon H′ index to represent realised plant diversity. The SEMs were conducted using the R package ‘*piecewiseSEM*’ (Lefcheck 2016). In order to test whether path sizes varied statistically between different levels of trophic complexity, we constructed the same pathways and fitted them as a multigroup SEM using the R package ‘*lavann*’ (Rosseel 2012), with each trophic complexity level as a group. By comparing a model in which all paths were fully unconstrained (were allowed to vary between groups) with models where single paths between single groups were constrained with ANOVA, we tested whether those paths statistically differed among the different levels of trophic complexity (low, moderate, high). Since the results from the *lavaan* and *piecewise* SEMs were qualitatively similar, we present the *piecewise* model results, as this approach accounts for the nested experimental structure with random effects.

To test whether the diversity–stability relationship changed across timescales of consumer manipulation, we calculated stability metrics for three different periods: 5‐year (2009–2013), 10‐year (2009–2018) and 15‐year (2009–2023) intervals. We then applied all the above analyses across these periods to test the consistency of our findings. Most of the results were consistent with timescale; therefore, we primarily present findings based on the 15‐year interval in the main text, unless otherwise noted. All analyses were programmed in R v 4.2.3 (R Development Core Team 2023).

## 3 Results

### 3.1 Effects of Biological Complexity on Diversity and Stability of Plant Communities

An increasing number of plant species consistently increased realised plant diversity (i.e., the Effective Number of Species (${\mathrm{ENS}}_{\mathrm{PIE}}$)), such as the inverse of the Simpson index and Shannon H′ index (Table S1, all cases *p* < 0.0001). Due to the mathematical relationships between richness, evenness, and these diversity metrics, increasing plant species richness decreased plant evenness (Table S1, all cases *p* < 0.0001). In contrast, simplifying trophic complexity, achieved by reducing heterotrophic group diversity, did not change the inverse of the Simpson index, Shannon H′ index or plant evenness over the 15‐year period (Table S1, all cases *p* > 0.05). Additionally, no interactive effects between plant species richness and trophic complexity dimensions were observed on these diversity indices (Table S1, all cases *p* > 0.05).

**FIGURE 2:**
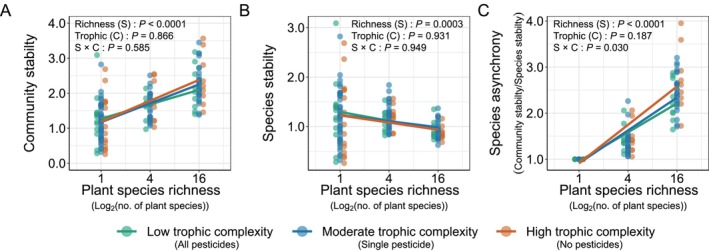
Effects of trophic complexity on the diversity−stability relationships in plant communities. Shown are changes in the diversity−community stability relationships (A) and its constituents: The diversity−species stability relationships (B) and the diversity−species asychonry relationships (C), across three trophic complexities. Plant species richness represents experimental biodiversity gradients, exemplified by treatments of 1, 4 and 16 sown plant species. The trophic complexity denotes the number of heterotrophic groups being reduced. We employed foliar fungicide, insecticide, and soil fungicide to target specific heterotrophic groups for reduction. In a low‐complexity setting, all pesticides were applied to reduce all three groups; in a moderate‐complexity scenario, only one group was reduced; and in a high‐complexity context, no heterotrophic groups were reduced. Data points represent values for each plot within the respective trophic complexity treatments (low: *N* = 32; moderate: *N* = 95; high: *N* = 33). Trend lines displayed represent simple asymptotic functions fitted to plant species richness at different trophic complexity treatments. Information about the model fit and sensitivity analyses using relatively shorter temporal data (5 years and 10 years) is provided in Table S2.

Increasing plant species richness increased the temporal mean and standard deviation of plant community biomass (Figures S2, S3 and Tables S1, S2, all cases *p* < 0.0001). This also led to increased community stability (Figure 2A and Table S2, *F*_*1,154*_ = 91.40, *p* < 0.0001), because the biomass mean increased by nearly twice as much as the variance. While reducing trophic complexity from high to low complexity increased the biomass mean by 24.4% ± 2.0% and standard deviation by 32.5% ± 1.8% (Figure S3 and Table S2, *F*_*2,154*_ = 3.24, *p* = 0.042 and *F*_*2,154*_ = 5.41, *p* = 0.005, respectively), trophic complexity reduction did not alter community stability (Figure 2A and Table S2, *F*_*2,154*_ = 0.14, *p* = 0.866). Reduced trophic complexity also had no interactive effects with plant species richness on plant community biomass (Figure S2 and Table S1, *F*_*2,164*_ = 0.99, *p* = 0.738), its temporal mean and standard deviation (Figure S3 and Table S2, *F*_*2,154*_ = 1.22, *p* = 0.300 and *F*_*2,154*_ = 1.74, *p* = 0.178, respectively) or community stability (Figure 2A and Table S2, *F*_*2,154*_ = 0.54, *p* = 0.585). These findings were largely consistent when analysed over shorter intervals of 10 years (2009–2018) or 5 years (2009–2013) (Table S1 − S2).

At the population level, increasing plant species richness reduced species stability (Figure 2B and Table S2, *F*_*1,154*_ = 13.73, *p* = 0.0003) consistently across levels of trophic complexity (Figure 2B and Table S2, *F*_*2,154*_ = 0.07, *p* = 0.932). Increasing plant species richness also increased species asynchrony (Figure 2C and Table S2, *F*_*1,154*_ = 707.36, *p* < 0.0001); this effect was more pronounced at high trophic complexity (e.g., 19.4% ± 1.4% higher in 16 plant species treatment), compared with low trophic complexity (Figure 2C and Table S2, *F*_*2,154*_ = 3.60, *p* = 0.030). However, the increase in species asynchrony driven by high trophic complexity was detected only over the longer 15‐year interval (2009–2023) but was not detected at early stages of the experiment (2009–2018, *p* = 0.335 and 2009–2013, *p* = 0.586).

### 3.2 Trophic Complexity Strengthens the Diversity–Asynchrony Relationship

**FIGURE 3:**
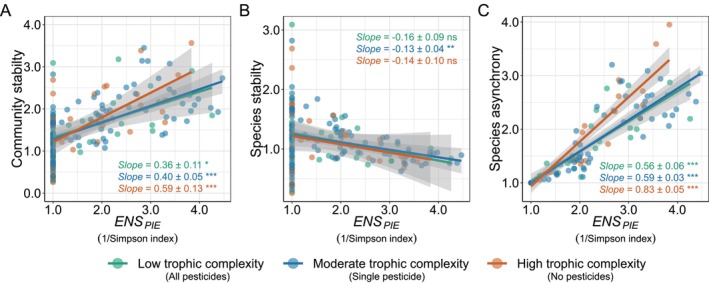
Changes in empirical relationships between realised plant diversity and stability of plant communities across trophic complexity. Note that the inverse of the Simpson index is used to represent the realised plant diversity, for example, the effective number of species (${\mathrm{ENS}}_{\mathrm{PIE}}$), in these models. Shown are the empirical relationship of ${\mathrm{ENS}}_{\mathrm{PIE}}$ with community stability (A), species stability (B) and specie asynchrony (C) at three different levels of trophic complexity. Data points represent values for each plot within the respective trophic complexity treatments (low: *N* = 32; moderate: *N* = 95; high: *N* = 33). The lines illustrate the trends fitted by linear models, encompassing 95% confidence intervals (shaded areas). Slopes represent the estimates and standard errors from the models. Significance levels: Ns: *P* > 0.05; *P ≤ 0.05; **P ≤ 0.001; ***P ≤ 0.0001. Details regarding the model fit, using Shannon H′ as ${\mathrm{ENS}}_{\mathrm{PIE}}$, and further sensitivity analyses using relatively shorter temporal data (5 years and 10 years) are provided in Tables S3–S5.

We found that the positive relationship between realised diversity measured as ${\mathrm{ENS}}_{\mathrm{PIE}}$ and community stability (Figure 3A and Table S3, *F*_*1,154*_ = 1341.83, *p* < 0.0001), as well as the negative relationship between ${\mathrm{ENS}}_{\mathrm{PIE}}$ and species stability (Figure 3B and Table S3, *F*_*1,154*_ = 726.98, *p* < 0.0001), remained consistent across varying levels of trophic complexity (community stability: *F*_*2,154*_ = 1.44, *p* = 0.239; species stability: *F*_*2,154*_ = 0.05, *p* = 0.914). However, we found a positive relationship between ${\mathrm{ENS}}_{\mathrm{PIE}}$ and species asynchrony (Figure 3C and Table S3, *F*_*1,154*_ = 5398.57, *p* < 0.0001), which was stronger under high trophic complexity (*F*_*2,154*_ = 9.05, *p* = 0.0002). We observed a consistent decrease in both the slopes and the variance explained by ${\mathrm{ENS}}_{\mathrm{PIE}}$ in relation to both species asynchrony and community stability across reducing levels of trophic complexity (Figure S3 and Table S3). Specifically, species asynchrony accounted for roughly twice the variance in community stability compared to species stability (Figure S4A,B and Table S3, species asynchrony: *R* ^*2*^: 0.41–0.54; species stability: *R* ^*2*^: 0.16–0.28). Also, the influence on changes in community stability changes attributed to the temporal mean of plant biomass (Figure S4C and Table S3, *R* ^*2*^: 0.47–0.53) was approximately three times greater than that of the standard deviation of plant biomass (Figure S4C and Table S3, *R* ^*2*^: 0.11–0.23). Additionally, the positive relationships between ${\mathrm{ENS}}_{\mathrm{PIE}}$ and both the temporal mean and standard deviation of plant biomass remained consistent with different levels of trophic complexity (Figure S5). These findings remained unchanged when using the Shannon H′ index and analysing the shorter intervals of 10 years (2009–2018) or 5 years (2009–2013) (Figures S5, S6 and Tables S4, S5).

**FIGURE 4:**
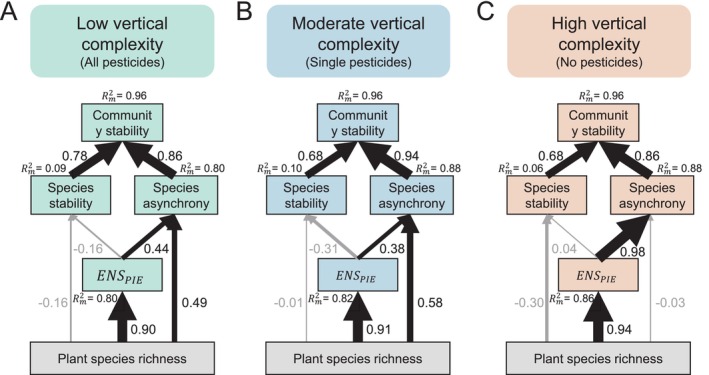
Trophic complexity mediating the direct and indirect effects of plant diversity on community stability. Shown are structural equation models (SEMs) that illustrate how plant species richness (i.e., increasing the number of plant species), through both direct impacts and indirectly by increasing realised plant diversity (${\mathrm{ENS}}_{\mathrm{PIE}}$), influences species stability and species asynchrony, which in turn balance plant community stability at three different levels of trophic complexity. Note that the inverse of the Simpson index is used to represent realised plant diversity, for example, the effective number of species (${\mathrm{ENS}}_{\mathrm{PIE}}$), in these SEMs. Low trophic complexity (A): Fisher's C = 1.439; df = 6; *p* = 0.963; AIC = 112.508; *N* = 32; moderate trophic complexity (B): Fisher's C = 9.123; df = 6; *p* = 0.167; AIC = 151.479; *N* = 95; high trophic complexity (C): Fisher's C = 3.808; df = 6; *p* = 0.703; AIC = 119.201; *N* = 33). Numbers represent standardised path coefficients; black indicate significant positive effects (*p* ≤ 0.05), while grey denote statistically nonsignificant effects (*p* > 0.05). The proportion of variance ($R_{m}^{2}$) explained by fixed factors in the model. Details regarding the statistics of the models, using Shannon H′ as ${\mathrm{ENS}}_{\mathrm{PIE}}$, and further sensitivity analyses using relatively shorter temporal data (5 years and 10 years) is provided Tables S6−S12.

Structural equation models (SEMs) revealed that in the low trophic complexity treatment (all heterotrophic groups reduced), increasing plant species richness directly increased both ${\mathrm{ENS}}_{\mathrm{PIE}}$ (Figure 4A, standardised path coefficients (SPEs) = 0.90, *p* < 0.0001) and species asynchrony (total direct SPEs = 0.49, *p* = 0.011). Additionally, the path from ${\mathrm{ENS}}_{\mathrm{PIE}}$ to species asynchrony was significant (SPEs = 0.44, *p* = 0.023), implying a predominance of direct effects over indirect ones (Figure 4A, total indirect SPEs = 0.39). Except for the difference in the magnitude of the SPEs, similar patterns were also observed in plots with moderate trophic complexity (Figure 4B). However, in high trophic complexity plots, where the entire spectrum of heterotrophic groups was intact, increasing plant species richness indirectly increased species asynchrony by first enhancing ${\mathrm{ENS}}_{\mathrm{PIE}}$ (Figure 4C, total indirect SPEs = 0.92), instead of exerting a direct effect on species asynchrony (total direct SPEs = −0.03, *p* = 0.848). Also, the pathway from ${\mathrm{ENS}}_{\mathrm{PIE}}$ to species asynchrony was stronger (SPEs = 0.98, *p* < 0.0001). Additional analyses using Shannon H′ (Figure S7), shorter intervals of 10 years (2009–2018) or 5 years (2009–2013), and multigroup SEMs all produced consistent results (Tables S6–S15).

## 4 Discussion

Ecological communities are organised into intricate networks of interactions among a diversity of species. These interactions span a wide range of relationships within and among trophic levels, including but not limited to predation, infection, competition and mutualism (Elton 1958). The net effect of these interactions on population, community or ecosystem stability has been a long‐standing debate (MacArthur 1955; May 1972; McNaughton 1978; McCann 2000; Worm and Duffy 2003; Hooper et al. 2005; Ives and Carpenter 2007; Tilman et al. 2014). While positive relationships between plant diversity and community stability have been well‐documented, the explanations have generally neglected to consider the potentially critical role of consumers. By manipulating both the number of plant species and the number of heterotrophic groups, we investigated the effects of multidimensional complexity on plant community stability and its constituents. Increasing plant species richness reduced species stability but increased community stability. Conversely, reducing the diversity of both plants and heterotrophs—a simplification of complexity within and among trophic levels—destabilised plant community biomass (Figure 2). Such destabilisation arose from increased synchronisation among the remaining plant species. Loss of heterotrophic diversity can amplify the synchronisation, potentially exacerbating the destabilisation of plant community biomass (Figures 3, 4). Our findings highlight the importance of diverse heterotrophic communities for maintaining plant community stability. However, we add the caveat that additional work in naturally assembling communities will aid the translation of these insights from biodiversity studies to more complex, multitrophic systems.

Our findings are consistent with previous diversity–stability studies focused on just the plant community that find greater stability in diverse plant communities compared to less diverse ones (McCann 2000; Hooper et al. 2005; Tilman et al. 2014; Loreau et al. 2021). However, greater plant diversity is often associated with greater trophic complexity (Cardinale et al. 2006; Haddad et al. 2009; Scherber et al. 2010; Li et al. 2023) and food‐web stability (Ebeling et al. 2008; Haddad et al. 2011; Borer et al. 2012). Our results, while consistent with earlier findings, advance understanding by mechanistically demonstrating that a reduction in trophic complexity destabilises plant communities due to increased synchronisation among remaining plant species (Figure 2). Increasing plant diversity has been shown to support enhanced ecosystem functions across trophic levels (Lefcheck et al. 2015; Soliveres et al. 2016; Barnes et al. 2020), and the current work adds to this the importance of heterotrophs for maintaining plant biomass stability. Increased synchronisation by reducing trophic complexity was more pronounced in diverse plant communities compared to simpler ones (Figure 2C), supporting the consumer–stabilisation hypothesis (Figure 1). This effect likely stems from greater variation in plant phenological traits in species‐rich communities. For example, reductions in consumers can slow plant senescence and delay the peak of greenness (Zaret et al. 2022). Heterotroph removal may also delay plant growth onset, leading to more synchronised growth across species, potentially linked to seasonal shifts in plant tissue carbon‐to‐nitrogen ratios (Ritchie et al. 1998; Sitters and Venterink 2015). Although reduced trophic complexity alters plant species' relative abundances (Borer et al. 2015; Seabloom et al. 2017), the hypothesis that asynchrony drives stability was supported only in the full 15‐year data set and not in shorter‐term observations (Table S2; Kohli et al. 2019). This discrepancy likely arises from the inability of shorter‐term studies to detect long‐term dynamics such as consumer–plant feedbacks, lagged effects, coevolution or gradual competitive exclusion. Additionally, short‐term studies can lack sufficient environmental variability to reveal how interspecific interactions affect stability (Tilman 1999). Theoretically, asynchrony in species growth rates, rather than abundances, may better capture short‐term trophic interactions (Loreau and de Mazancourt 2008). Longer‐term studies across variable environmental conditions are needed to reveal these dynamics.

Community stability increased with the number of plant species largely due to increased species asynchrony, rather than increased species stability (Figures 3 and 4). This pattern is also well‐documented in theoretical (Tilman 1999; Lehman and Tilman 2000; Loreau and de Mazancourt 2008, 2013) and empirical studies (Hector et al. 2010; Hautier et al. 2014; Craven et al. 2018; Schnabel et al. 2021; Liang et al. 2022). Intriguingly, our findings further reveal that trophic complexity has the potential to modulate the direct and indirect effects of plant species richness on community stability and its associated components. While there was a strong link between plant species richness and realised plant diversity (e.g., effective number of species) at all levels of trophic complexity, there was a shift from direct effects of plant species richness on species asynchrony (Figure 4A, *Plant species richness* → *species asynchrony*) to indirect effects mediated by realised plant diversity (Figure 4C, *Plant species richness* → ${\mathrm{ENS}}_{\mathrm{PIE}}$ → *species asynchrony*) with increasing trophic complexity. Ultimately, reductions in indirect effects caused by the loss of trophic diversity weakened the associations between realised plant diversity and species asynchrony, which can potentially lead to lower plant community stability. This might be because the loss of diverse heterotrophic groups causes increased interspecific competition among plant species, which in turn could synchronise plant population dynamics and contribute to destabilisation (Ives et al. 1999; Loreau and de Mazancourt 2008, 2013; Thibaut and Connolly 2013). However, should the heterotrophic population's consumptive intensity surpass the capacity of the plants to sustain, it could result in the overall destabilisation of plant communities through ‘overgrazing’ (Rakowski and Cardinale 2016; Liang et al. 2021), regardless of the direct and indirect shifts observed in our study. In natural systems, vertebrate herbivores (e.g., deer, rodents) may overshadow these mechanisms, highlighting the need to integrate experimental and observational studies.

Our findings in terrestrial grasslands contrast with previous meta‐analyses, which have primarily focused on aquatic multitrophic systems and largely inferred from correlation analyses. While we observed a negative relationship between plant diversity and species stability, prior meta‐analyses have reported a positive diversity–stability relationship in aquatic systems that are characterised by higher trophic complexity (Jiang and Pu 2009; Xu et al. 2021). For example, kelp forests exhibited a positive diversity–stability relationship for understory algae but no effect on species asynchrony (Lamy et al. 2020; but see Liang et al. 2024). Aquatic systems, driven by strong top‐down interactions involving fish and invertebrates (Shurin et al. 2002), may be stabilised by a shift to weaker trophic interactions (McCann et al. 1998; Jiang and Pu 2009; Xu et al. 2021). In contrast, the grassland communities of this experiment exhibited relatively low consumptive intensity, with arthropods consuming only 20% of plant biomass (Figure S3A). Nonetheless, increased species asynchrony contributed to greater stability in plant communities, consistent with Zhao et al. (2019). These results suggest that weak trophic interactions in grasslands may impact stability differently than in aquatic systems. Instead, our findings highlight that plant–consumer interactions modulate community stability indirectly via synchronisation of plant population dynamics. These differences suggest that the facets of trophic complexity, including trophic level lengths, interaction strengths and predators, may further shape complexity–stability relationships (Thébault and Loreau 2005; Zhao et al. 2019; Firkowski et al. 2021; Eschenbrenner and Thébault 2023).

Biodiversity loss is increasing across the tree of life as a result of global changes (Sala et al. 2000), with higher trophic levels experiencing more severe losses (Hallmann et al. 2017; van Klink et al. 2020). Yet, much of the previous research on the diversity–stability relationship has primarily concentrated on primary producers because of their fundamental role as the source of food and energy for higher trophic groups. Our study expands this research scope to include trophic complexity across different consumer groups. Notably, we found that, independent of consumer group identity, consumer diversity can strengthen the link between plant diversity and community stability due to reduced synchronisation among plant species. These relationships are particularly apparent in long‐term (15‐year period) system dynamics. Trophic complexity may thus stabilise ecosystems in the face of environmental perturbations. Conserving consumer biodiversity while maintaining complexity across various dimensions of species interactions will maintain both biodiversity and the stable functioning of ecosystems.

## Author Contributions

M.L. conceived the idea, analysed the data and wrote the first draft. D.T., E.T.B. and E.W.S. designed the experiment. All authors contributed to the development of the study.

### Peer Review

The peer review history for this article is available at https://www.webofscience.com/api/gateway/wos/peer‐review/10.1111/ele.70103.

## Supporting information


Data S1.

## Data Availability

Original data sets are available at the Environmental Data Initiative (https://doi.org/10.6073/pasta/d35065bdbebf73b310a21815616f66b2). All code and data used in our study are publicly available on zenodo (https://doi.org/10.5281/zenodo.14602463).
