# Supplementary material for: Consumers Modulate Effects of Plant Diversity on Community Stability
Source: Ecol Lett. 2025 Mar 20;28(3):e70103. doi: 10.1111/ele.70103 (PMC11924315; doi:10.1111/ele.70103)
Supplement: Supplementary file 1 — Data S1. [file ELE-28-0-s001.docx]

**Supplementary Material**

***for***

**Consumers Modulate Effects of Plant Diversity on Community Stability**

Maowei Liang^1*^, ​Seraina L. Cappelli^2^, Elizabeth T. Borer^2^, David Tilman^2^, Eric W. Seabloom^2^

***Correspondence author:** Maowei Liang, E-mail: [mwliang@umn.edu](mailto:mwliang@umn.edu);

**This file includes:**

**Figures S1–S7;**

**Tables S1–S15.**

**
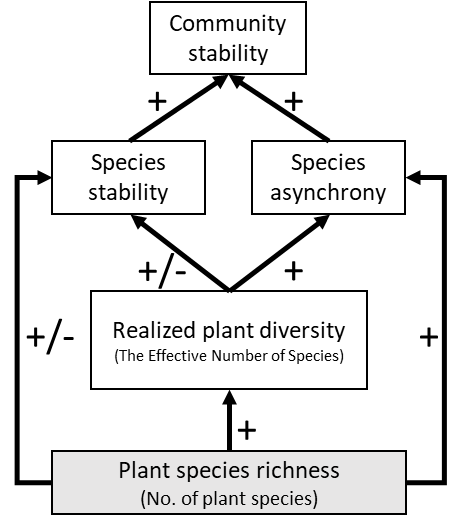
**

| **NO.** | **Associations** | **Rationale** |
| --- | --- | --- |
| Ⅰ. *The direct effects of plant species richness on diversity metrics* | | |
| **1** | **Plant species richness → Plant diversity** | In general, increasing a number of plant species will increase realized plant diversity (e.g., invers Simpson and Shannon H’ indices). |
| Ⅱ. *The direct effects of plant richness on stability metrics* | | |
| **2** | **Plant species richness → Species stability** | Increasing a variety of plant species tends to decrease species stability (Tilman *et al.* 2006) while increasing species asynchrony (Hector *et al.* 2010, Schnabel *et al.* 2021; Wagg *et al.* 2022). |
| **3** | **Plant species richness → Species asynchrony** |  |
| Ⅲ. *The direct effects of diversity on stability metrics* | | |
| **4** | **Plant diversity → Species stability** | Plant diversity can both decrease and increases species stability (Thibaut and Connolly 2013) |
| **5** | **Plant diversity → Species asynchrony** | It has been well documented that plant diversity increases species asynchrony (Hautier *et al.* 2014, Craven *et al.* 2018, Liang *et al.* 2022). |
| Ⅳ. *The relationships between species stability/species asynchrony and community stability* | | |
| **6** | **Species stability → Community stability** | Community stability of plant biomass can be mathematically partitioned into the product of species stability (i.e., the weighted average of local species stability) and species asynchrony among plant species (Loreau and de Mazancourt 2008; Thibaut and Connolly 2013; Wang *et al.* 2019). Therefore, both species stability and species asynchrony are positively related to community stability and together fully explain its variations. |
| **7** | **Species asynchrony → Community stability** |  |

**Figure S1 A hypothesized structural equation modeling (SEM) illustrating the direct and indirect effects of plant species richness (i.e., no. of plant species) on community stability and its constituents by changing realized plant diversity (hereafter, plant diversity).**


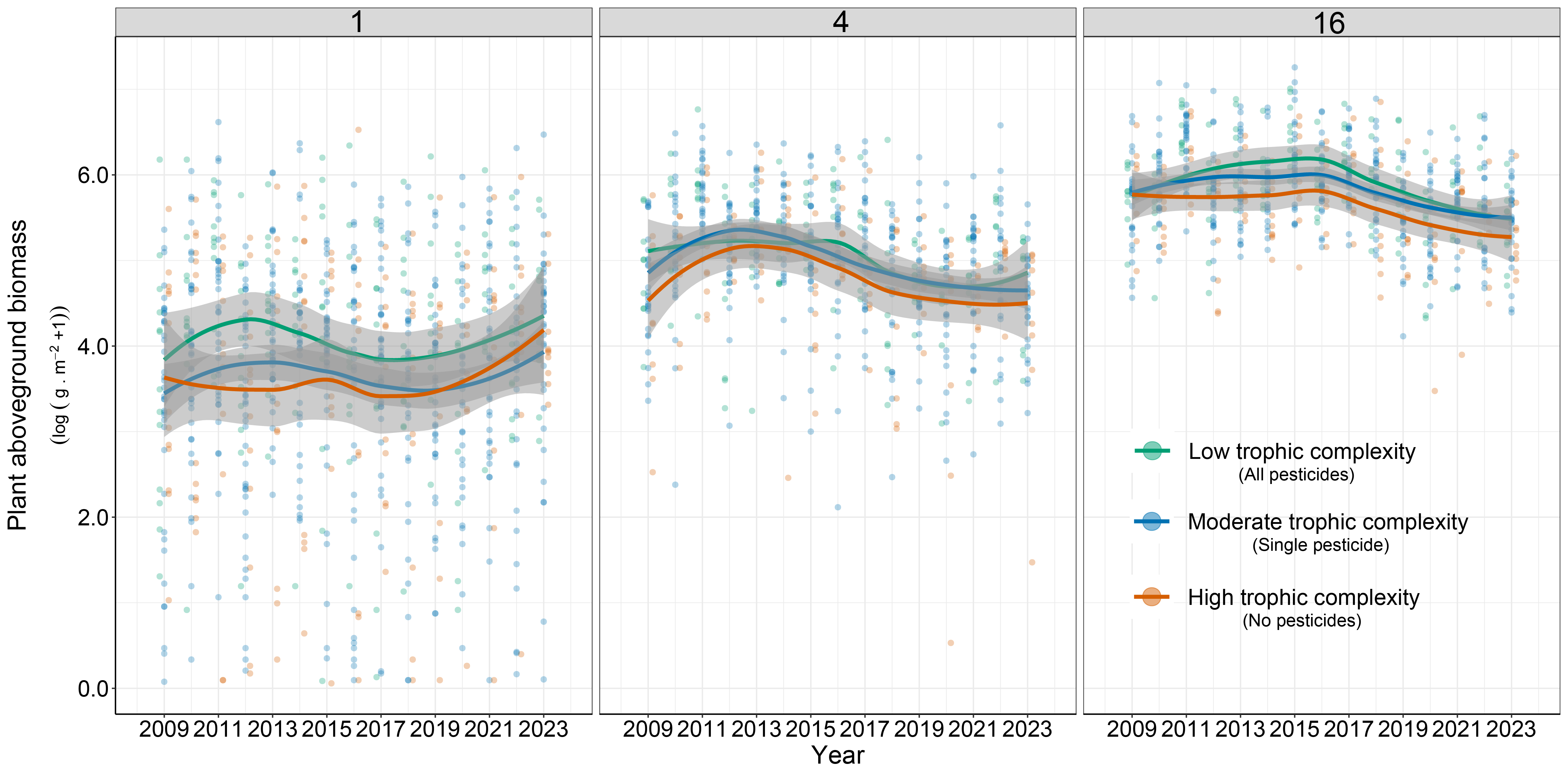


**Figure S2 Decadal-long changes in plant community biomass across biological** **complexities.** Plant species richness represents experimental biodiversity gradients, exemplified by treatments of 1, 4, and 16 sown plant species. The trophic complexity denotes the number of heterotrophic groups being reduced. We employed foliar fungicide, insecticide, and soil fungicide to target specific heterotrophic groups for reduction. In a low-complexity setting, all pesticides were applied to reduce all three groups; in a moderate-complexity scenario, only one group was reduced; and in a high-complexity context, no heterotrophic groups were reduced. Data points represent values for each plot within the respective trophic complexity treatments (low: N = 32; moderate: N = 95; high: N = 33). Trend lines displayed represent the long-term mean of plant community biomass, encompassing 95% confidence intervals (shaded areas). Results of three-way ANOVA (plant species richness, trophic complexity, and year) are provided in Table S1.


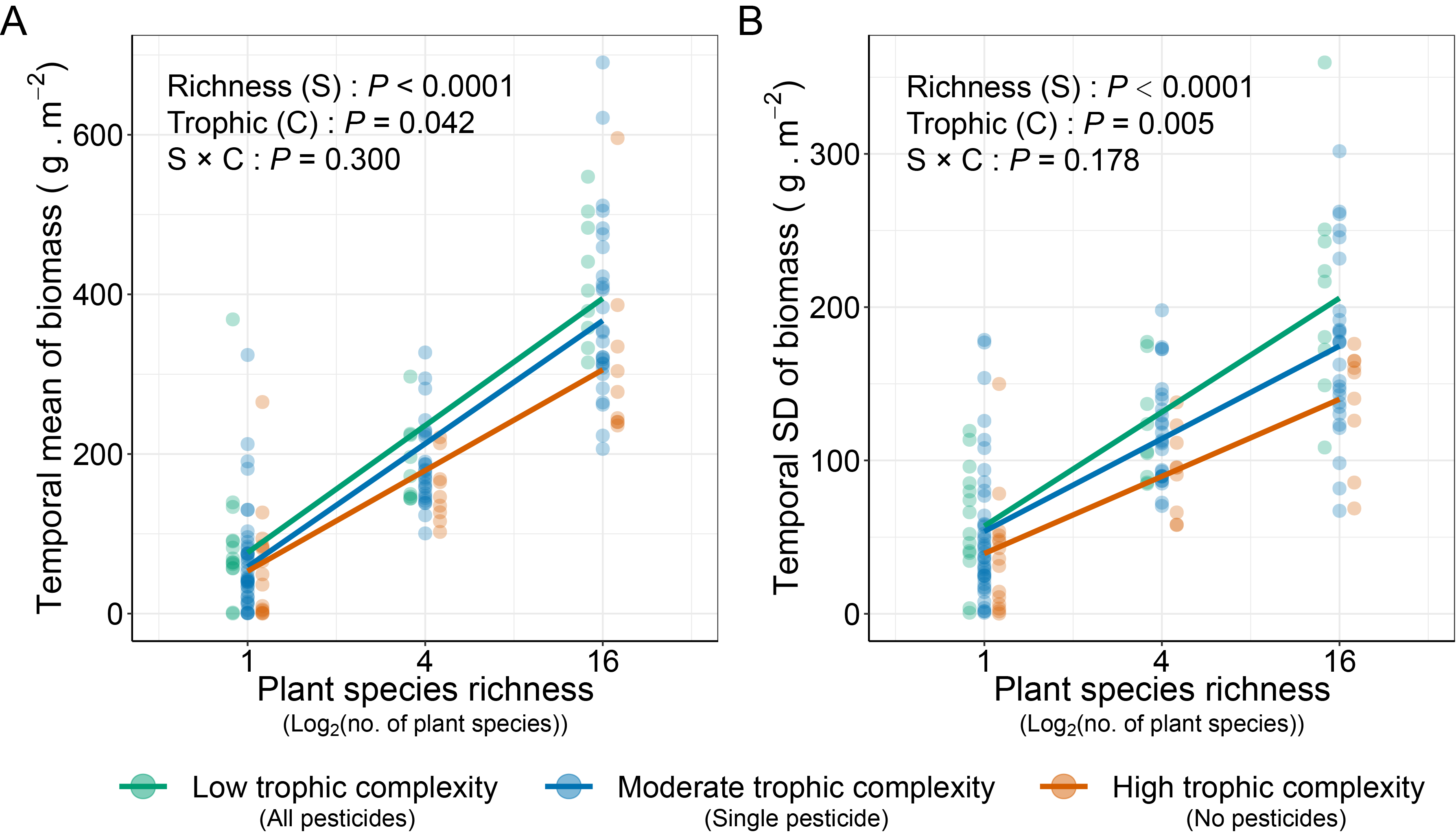


**Figure S3 Effects of trophic complexity on the temporal mean and standard deviation** **of plant community biomass.** Shown are changes in the temporal mean (A) and standard deviation (B) of plant community biomass along both plant species richness and trophic complexity gradients. Plant species richness represents experimental biodiversity gradients, exemplified by treatments of 1, 4, and 16 sown plant species. The trophic complexity denotes the number of heterotrophic groups being reduced. We employed foliar fungicide, insecticide, and soil fungicide to target specific heterotrophic groups for reduction. In a low-complexity setting, all pesticides were applied to reduce all three groups; in a moderate-complexity scenario, only one group was reduced; and in a high-complexity context, no heterotrophic groups were reduced. Data points represent values for each plot within the respective trophic complexity treatments (low: N = 32; moderate: N = 95; high: N = 33). Trend lines displayed represent simple asymptotic functions fitted to plant species richness at different trophic complexity treatments. Information about the model fit is provided in Table S2.


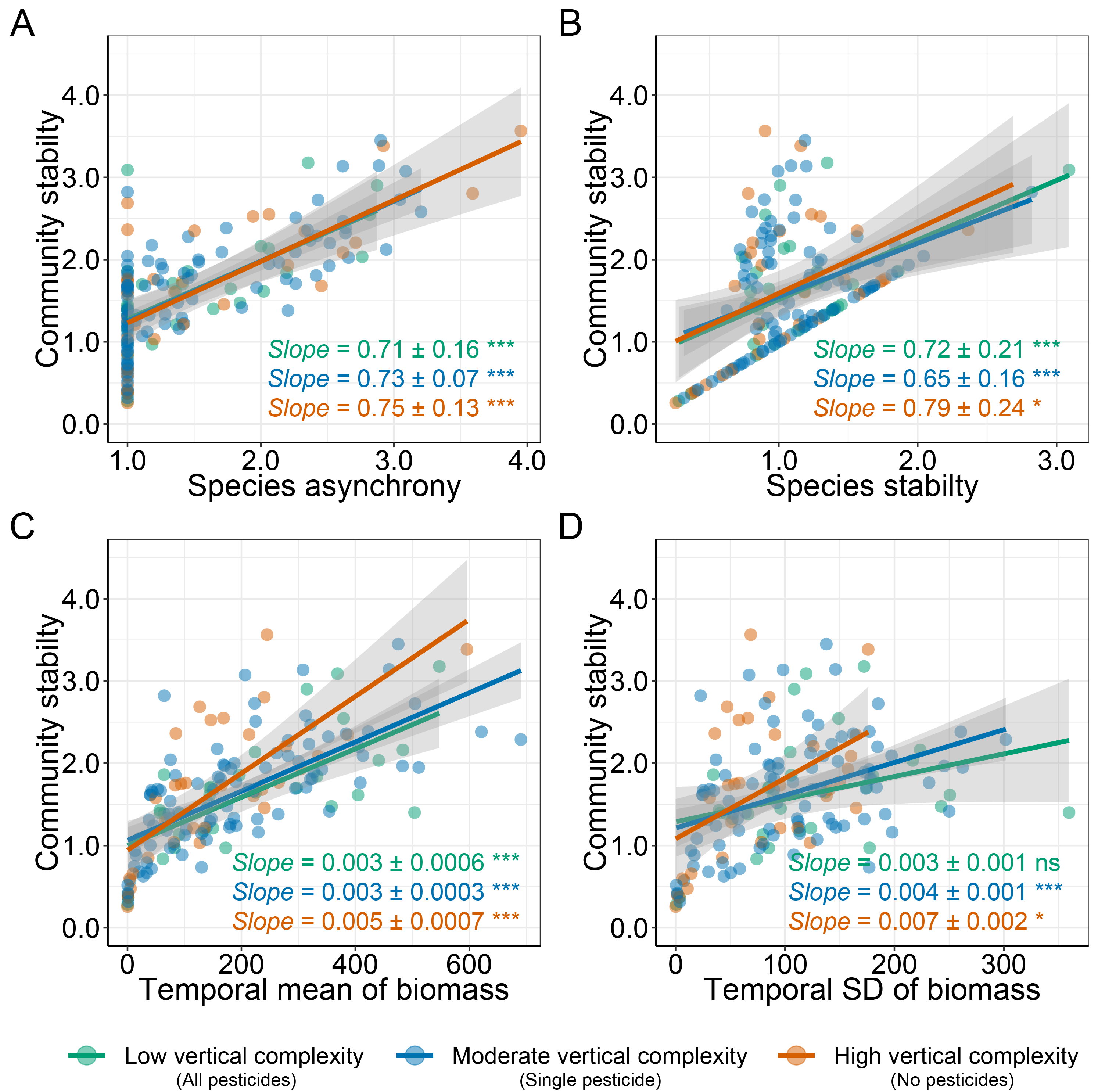


**Figure S4 Empirical relationships between plant community stability and its constituents across trophic complexity.** Shown are the empirical relationship of community stability with specie asynchrony (A), species stability (B), the temporal mean of biomass (C), and the temporal standard deviation of biomass (D) at three different levels of trophic complexity. Data points represent values for each plot within the respective trophic complexity treatments (low: N = 32; moderate: N = 95; high: N = 33). The lines illustrate the trends fitted by linear models, encompassing 95% confidence intervals (shaded areas). Slopes represent the estimates and standard errors from the models. Significance levels: ns: *P* > 0.05; *: *P* ≤ 0.05; **: *P* ≤ 0.001; ***: *P* ≤ 0.0001. Information about the model fit is provided in Table S3.


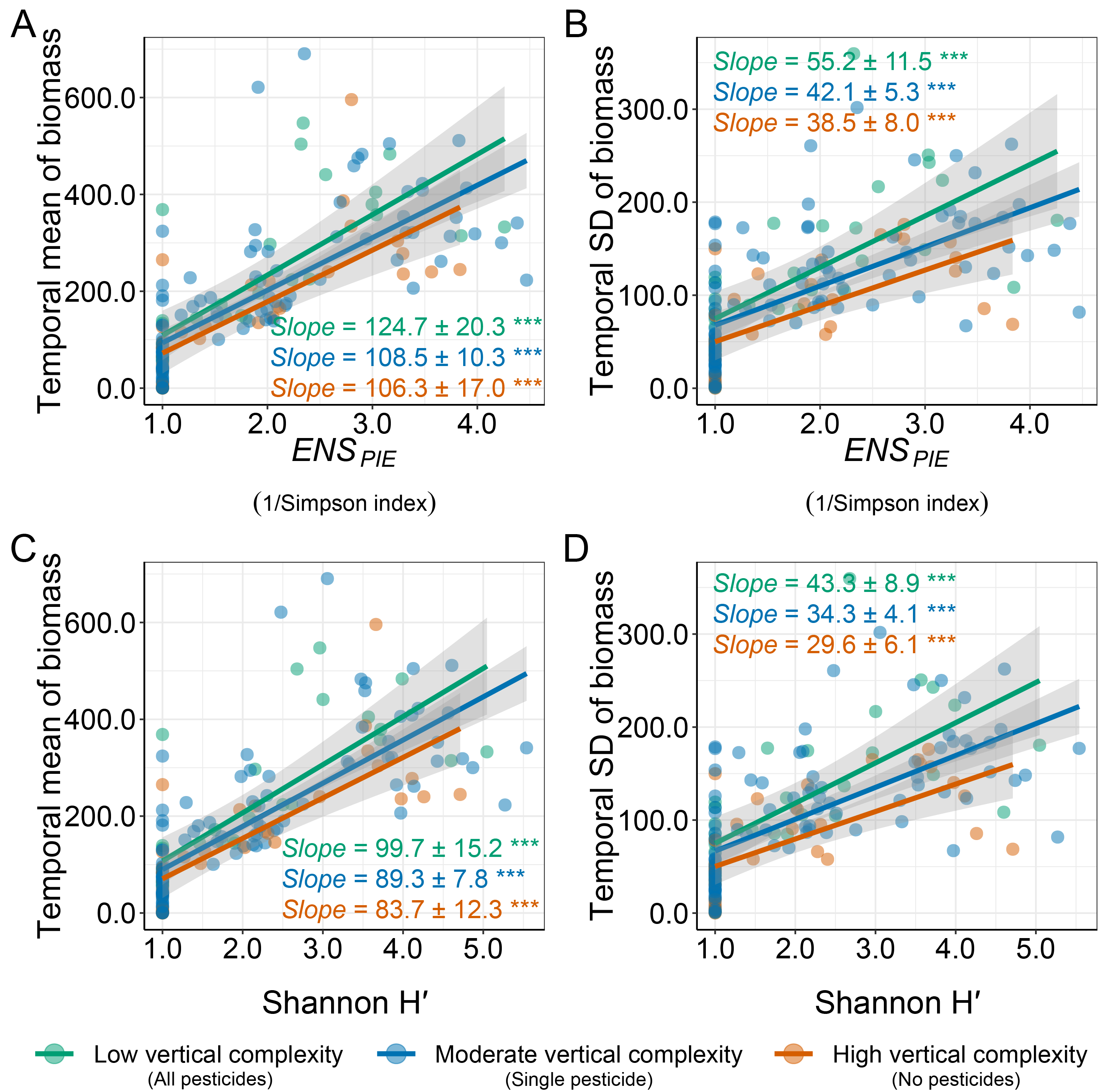


**Figure S5 Empirical relationships between realized plant** **diversity and temporal mean and standard deviation** **of plant community biomass across trophic complexity.** Shown are the empirical relationship of realized plant diversity to temporal mean and standard deviation of biomass at three different levels of trophic complexity. Both the inverse of the Simpson index (A and B, ${ENS}_{PIE}$) and Shannon H' index (C and D) are used to robustly represent plant diversity in these models. Data points represent values for each plot within the respective trophic complexity treatments (low: N = 32; moderate: N = 95; high: N = 33). The lines illustrate the trends fitted by linear models, encompassing 95% confidence intervals (shaded areas). Slopes represent the estimates and standard errors from the models. Significance levels: ns: *P* > 0.05; *: *P* ≤ 0.05; **: *P* ≤ 0.001; ***: *P* ≤ 0.0001. Information about the model fit is provided in Table S3.


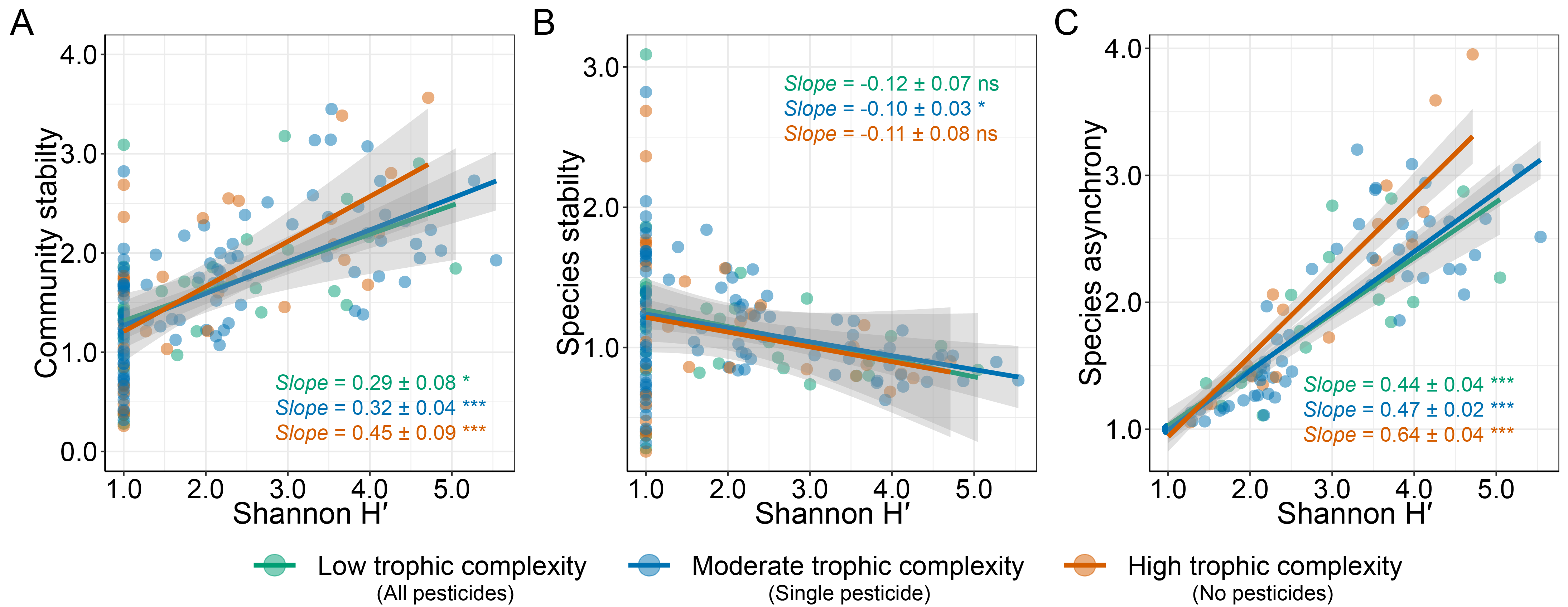


Figure S6 **Empirical relationships between realized plant** **diversity (Shannon H') and stability of plant communities across trophic complexity.** Shown are the empirical relationship of Shannon H' index to specie asynchrony (A), species stability (B), and community stability (C) at three different levels of trophic complexity. Data points represent values for each plot within the respective trophic complexity treatments (low: N = 32; moderate: N = 95; high: N = 33). The lines illustrate the trends fitted by linear models, encompassing 95% confidence intervals (shaded areas). Slopes represent the estimates and standard errors from the models. Significance levels: ns: *P* > 0.05; *: *P* ≤ 0.05; **: *P* ≤ 0.001; ***: *P* ≤ 0.0001. Note that the Shannon H' index is used to represent realized plant diversity in these models. Details regarding the model fit is provided in Table S3.


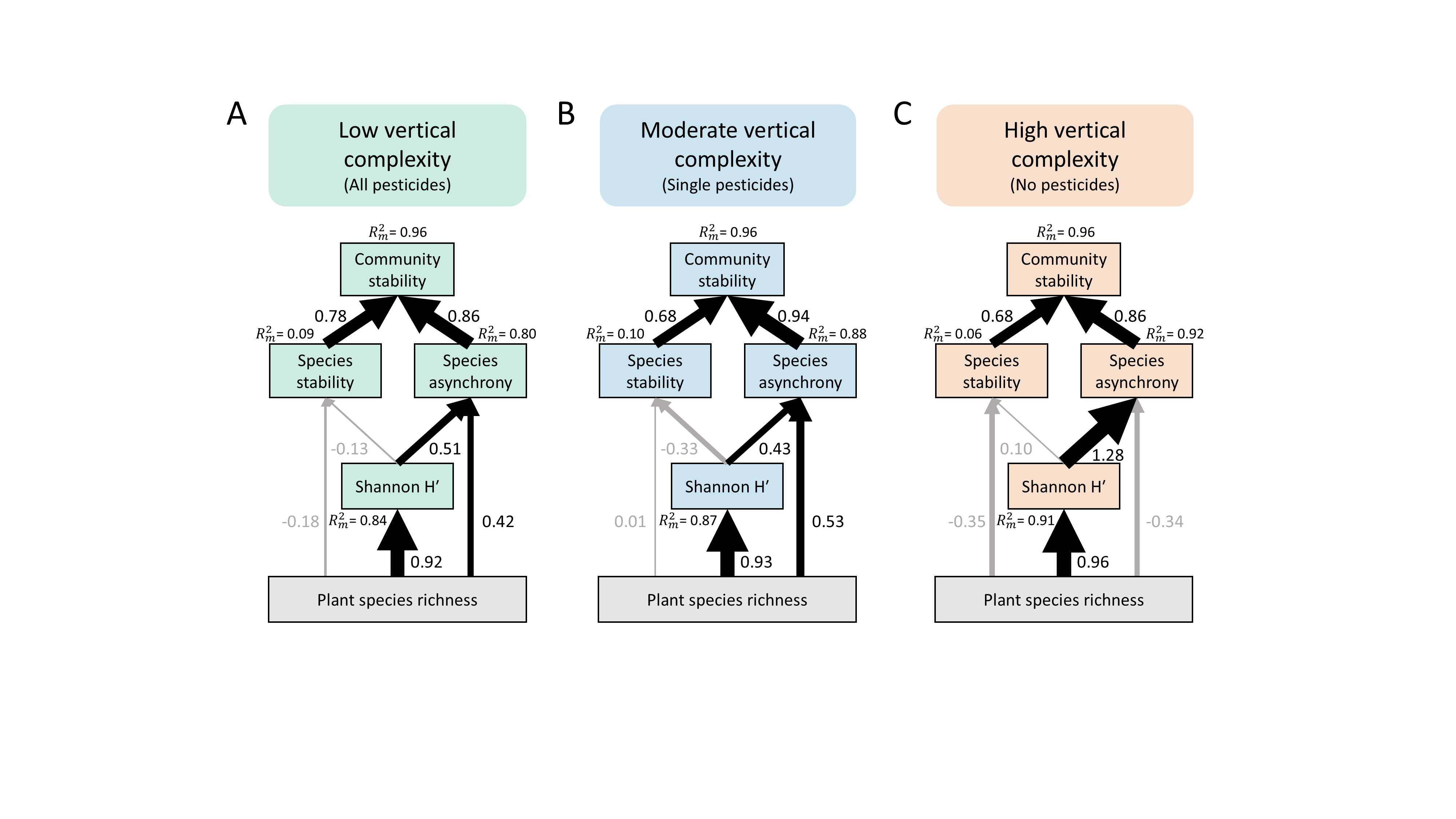


Figure S7 **Trophic** **complexity mediating the direct and indirect effects of plant richness on plant community stability by changing realized plant diversity (Shannon H').** Shown are structural equation models (SEMs) that illustrate how plant species richness (i.e., increasing the number of plant species), through both direct impacts and indirectly by increasing realized plant diversity (Shannon H'), influences species stability and species asynchrony, which in turn balance plant community stability at three different levels of trophic complexity. Low (A): Fisher's C = 1.232; df = 6; p = 0.975; AIC = 121.129; N = 32; Moderate (B): Fisher's C = 7.938; df = 6; p = 0.243; AIC = 175.337; N = 95; High (C): Fisher's C = 4.730; df = 6; p = 0.579; AIC = 117.849; N = 33). Numbers represent standardized path coefficients; black indicate significant positive effects (*P* ≤ 0.05), while grey denotes statistically nonsignificant effects (*P* > 0.05). The proportion of variance ($R_{m}^{2}$) explained by fixed factors in the model. Note that the Shannon H' index is used to represent plant diversity in these SEMs. Information regarding the statistics of the models is provided Tables S9 and S13.

**Table S1** Results for the effects of plant species richness (i.e., number of plant species, S), trophic complexity (i.e., number of heterotrophic groups, C), year (Y), and their interactive forcing on the effective number of species (${ENS}_{PIE}$, 1/Simpson), evenness (${ENS}_{PIE}$/richness), Shannon H' index, and plant community biomass. Mixed effects models were used with ‘*plotID*’ as a random factor and incorporating a first-order autocorrelation structure, “*corAR1(form =   year | plotID)*”. The analysis was conducted for different periods: 15-year (2009−2023), 10-year (2009−2018), and 5-year (2009−2013) intervals, respectively. Significant effects (*P* ≤ 0.05) are shown in bold text.

|  | *df* | | ${ENS}_{PIE}$ (1/Simpson) | | Plant evenness  (${ENS}_{PIE}$/richness) | | Shannon index  (H') | | Plant biomass  (g/m^2^) | |
| --- | --- | --- | --- | --- | --- | --- | --- | --- | --- | --- |
|  | *numDF* | *denDF* | *F-values* | *P-values* | *F-values* | *P-values* | *F-values* | *P-values* | *F-values* | *P-values* |
| 15-year interval (2009−2023) | | | | | | | | | | |
| Intercept | *1* | *2081* | **3292.659** | **<0.0001** | **5637.708** | **<0.0001** | **3304.957** | **<0.0001** | **880.790** | **<0.0001** |
| Plant species richness (S) | *1* | *154* | **766.405** | **<0.0001** | **461.054** | **<0.0001** | **1082.178** | **<0.0001** | **355.511** | **<0.0001** |
| Trophic complexity (C) | *2* | *154* | 0.507 | 0.6036 | 0.001 | 0.9989 | 0.413 | 0.6622 | **3.167** | **0.0449** |
| Year (Y) | *1* | *2081* | **86.592** | **<0.0001** | **25.389** | **<0.0001** | **108.508** | **<0.0001** | **112.565** | **<0.0001** |
| S × C | *2* | *154* | 0.973 | 0.3804 | 0.115 | 0.8918 | 0.808 | 0.4477 | 1.241 | 0.2921 |
| S × Y | *1* | *2081* | **147.679** | **<0.0001** | **25.233** | **<0.0001** | **182.685** | **<0.0001** | **54.213** | **<0.0001** |
| C × Y | *2* | *2081* | 1.993 | 0.1365 | 0.534 | 0.5862 | 2.454 | 0.0862 | 0.283 | 0.7537 |
| S × C × Y | *2* | *2081* | **3.994** | **0.0186** | 1.182 | 0.3070 | **5.739** | **0.0033** | 0.225 | 0.7988 |
| 10-year interval (2009−2018) | | | | | | | | | | |
| Intercept | *1* | *1344* | **3324.235** | **<0.0001** | **5727.988** | **<0.0001** | **3441.197** | **<0.0001** | **914.096** | **<0.0001** |
| Plant species richness (S) | *1* | *154* | **875.320** | **<0.0001** | **427.619** | **<0.0001** | **1237.347** | **<0.0001** | **392.893** | **<0.0001** |
| Trophic complexity (C) | *2* | *154* | 0.761 | 0.4687 | 0.051 | 0.9505 | 0.720 | 0.4882 | **3.365** | **0.0371** |
| Year (Y) | *1* | *1344* | **32.470** | **<0.0001** | **15.592** | **0.0001** | **32.553** | **<0.0001** | 0.115 | 0.7349 |
| S × C | *2* | *154* | 1.467 | 0.2338 | 0.133 | 0.8752 | 1.308 | 0.2733 | 1.520 | 0.2219 |
| S × Y | *1* | *1344* | **61.050** | **<0.0001** | **10.221** | **0.0014** | **64.034** | **<0.0001** | **5.935** | **0.0150** |
| C × Y | *2* | *1344* | 0.701 | 0.4964 | 0.055 | 0.9460 | 0.845 | 0.4298 | 0.311 | 0.7332 |
| S × C × Y | *2* | *1344* | 2.256 | 0.1051 | 0.289 | 0.7490 | 2.065 | 0.1272 | 1.449 | 0.2352 |
| 5-year interval (2009−2013) | | | | | | | | | | |
| Intercept | *1* | 598 | **2961.676** | **<0.0001** | **5508.125** | **<0.0001** | **3242.609** | **<0.0001** | **813.603** | **<0.0001** |
| Plant species richness (S) | *1* | 154 | **864.043** | **<0.0001** | **369.647** | **<0.0001** | **1257.550** | **<0.0001** | **309.410** | **<0.0001** |
| Trophic complexity (C) | *2* | 154 | 1.003 | 0.3692 | 0.036 | 0.9646 | 1.107 | 0.3332 | 2.942 | 0.0557 |
| Year (Y) | *1* | 598 | 0.533 | 0.4657 | 0.002 | 0.9661 | 0.582 | 0.4457 | **10.875** | **0.0010** |
| S × C | *2* | 154 | 2.411 | 0.0931 | 0.200 | 0.8189 | 2.183 | 0.1162 | 0.747 | 0.4757 |
| S × Y | *1* | 598 | 0.315 | 0.5748 | 0.033 | 0.8554 | 0.174 | 0.6764 | 1.722 | 0.1899 |
| C × Y | *2* | 598 | 1.599 | 0.2030 | 2.116 | 0.1214 | 1.754 | 0.1740 | 0.284 | 0.7526 |
| S × C × Y | *2* | 598 | 1.876 | 0.1541 | 0.102 | 0.9031 | 2.772 | 0.0633 | **3.159** | **0.0432** |

**Table S2** Results for the effects of plant species richness (i.e., number of plant species, S), trophic complexity (i.e., number of heterotrophic groups, C), and their interactive forcing on community stability, species stability, species asynchrony, the temporal mean and standard deviation of plant community biomass. Mixed effects models were used with ‘*plotID*’ as a random factor. The analysis was conducted for different periods: 15-year (2009−2023), 10-year (2009−2018), and 5-year (2009−2013) intervals, respectively. Significant effects (*P* ≤ 0.05) are shown in bold text.

|  | Plant species richness (S) | | Trophic complexity (C) | | S × C | |
| --- | --- | --- | --- | --- | --- | --- |
|  | *F-values* | *P-values* | *F-values* | *P-values* | *F-values* | *P-values* |
| 15-year interval (2009−2023) | | | | | | |
| Community stability | **91.402** | **<0.0001** | 0.145 | 0.8656 | 0.538 | 0.5848 |
| Species stability | **13.734** | **0.0003** | 0.071 | 0.9318 | 0.053 | 0.9488 |
| Species asynchrony | **707.361** | **<0.0001** | 1.696 | 0.1867 | **3.601** | **0.0296** |
| Temporal mean of biomass | **366.752** | **<0.0001** | **3.244** | **0.0417** | 1.215 | 0.2995 |
| Temporal SD of biomass | **165.032** | **<0.0001** | **5.411** | **0.0054** | 1.744 | 0.1782 |
| 10-year interval (2009−2018) | | | | | | |
| Community stability | **114.499** | **<0.0001** | 0.055 | 0.9468 | 0.257 | 0.7737 |
| Species stability | **8.360** | **0.0044** | 0.167 | 0.8466 | 0.022 | 0.9782 |
| Species asynchrony | **487.674** | **<0.0001** | 0.157 | 0.8551 | 1.103 | 0.3345 |
| Temporal mean of biomass | **401.620** | **<0.0001** | **3.357** | **0.0374** | 1.371 | 0.2570 |
| Temporal SD of biomass | **132.163** | **<0.0001** | **3.610** | **0.0294** | 1.222 | 0.2976 |
| 5-year interval (2009−2013) | | | | | | |
| Community stability | **39.292** | **<0.0001** | 0.433 | 0.6491 | 0.263 | 0.7690 |
| Species stability | **7.734** | **0.0061** | 0.0001 | 0.9999 | 0.146 | 0.8645 |
| Species asynchrony | **159.641** | **<0.0001** | 0.877 | 0.4182 | 0.537 | 0.5856 |
| Temporal mean of biomass | **318.863** | **<0.0001** | 2.712 | 0.0696 | 0.692 | 0.5020 |
| Temporal SD of biomass | **106.439** | **<0.0001** | **3.567** | **0.0306** | 1.228 | 0.2957 |

**Table S3** Results for bivariate correlation analyses based on ordinary least squares linear regression models (LMs) were obtained for the interval between 2009 and 2023, within a 15-year period. These analyses were conducted at three distinct levels of trophic complexity (low: N = 32; moderate: N = 95; high: N = 33). *R^2^* is the explained variance in LMs. Bold face denotes *P* ≤ 0.05.

| NO. | Models | Trophic complexities | Estimate | Std. Error | *P-values* | *R^2^* |
| --- | --- | --- | --- | --- | --- | --- |
| 01 | Community stability ~ Species asynchrony | **Low** | **0.705** | **0.155** | **0.0001** | **0.407** |
|  |  | **Moderate** | **0.729** | **0.070** | **<0.0001** | **0.538** |
|  |  | **High** | **0.747** | **0.129** | **<0.0001** | **0.518** |
| 02 | Community stability ~ Species stability | **Low** | **0.724** | **0.214** | **0.0020** | **0.277** |
|  |  | **Moderate** | **0.648** | **0.155** | **0.0001** | **0.158** |
|  |  | **High** | **0.786** | **0.244** | **0.0030** | **0.250** |
| 03 | Community stability ~ Temporal mean of biomass | **Low** | **0.003** | **0.001** | **<0.0001** | **0.482** |
|  |  | **Moderate** | **0.003** | **0.0003** | **<0.0001** | **0.471** |
|  |  | **High** | **0.005** | **0.001** | **<0.0001** | **0.529** |
| 04 | Community stability ~ Temporal SD of biomass | Low | 0.003 | 0.001 | 0.0686 | 0.106 |
|  |  | **Moderate** | **0.004** | **0.001** | **<0.0001** | **0.168** |
|  |  | **High** | **0.007** | **0.002** | **0.0051** | **0.226** |
| 05 | Community stability ~ ${ENS}_{PIE}$ | **Low** | **0.362** | **0.111** | **0.0027** | **0.262** |
|  |  | **Moderate** | **0.396** | **0.052** | **<0.0001** | **0.381** |
|  |  | **High** | **0.587** | **0.126** | **0.0001** | **0.413** |
| 06 | Community stability ~ Shannon H' | **Low** | **0.291** | **0.085** | **0.0018** | **0.281** |
|  |  | **Moderate** | **0.321** | **0.041** | **<0.0001** | **0.399** |
|  |  | **High** | **0.454** | **0.094** | **<0.0001** | **0.428** |
| 07 | Species asynchrony ~ ${ENS}_{PIE}$ | **Low** | 0.559 | 0.057 | **<0.0001** | 0.764 |
|  |  | **Moderate** | 0.587 | 0.028 | **<0.0001** | 0.828 |
|  |  | **High** | 0.831 | 0.053 | **<0.0001** | 0.889 |
| 08 | Species asynchrony ~ Shannon H' | **Low** | 0.441 | 0.042 | **<0.0001** | 0.787 |
|  |  | **Moderate** | 0.470 | 0.021 | **<0.0001** | 0.846 |
|  |  | **High** | 0.639 | 0.036 | **<0.0001** | 0.911 |
| 09 | Species stability ~ ${ENS}_{PIE}$ | Low | -0.155 | 0.089 | 0.0925 | 0.091 |
|  |  | **Moderate** | **-0.126** | **0.039** | **0.0015** | **0.103** |
|  |  | High | -0.139 | 0.102 | 0.1810 | 0.057 |
| 10 | Species stability ~ Shannon H' | Low | -0.120 | 0.070 | 0.0961 | 0.090 |
|  |  | **Moderate** | **-0.099** | **0.031** | **0.0016** | **0.102** |
|  |  | High | -0.106 | 0.077 | 0.1804 | 0.057 |
| 11 | Temporal mean of biomass ~ ${ENS}_{PIE}$ | **Low** | **124.649** | **20.234** | **<0.0001** | **0.559** |
|  |  | **Moderate** | **108.485** | **10.341** | **<0.0001** | **0.542** |
|  |  | **High** | **106.320** | **16.951** | **<0.0001** | **0.559** |
| 12 | Temporal mean of biomass ~ Shannon H' | **Low** | **99.660** | **15.159** | **<0.0001** | **0.590** |
|  |  | **Moderate** | **89.324** | **7.776** | **<0.0001** | **0.587** |
|  |  | **High** | **83.717** | **12.256** | **<0.0001** | **0.601** |
| 13 | Temporal SD of biomass ~ ${ENS}_{PIE}$ | **Low** | **55.231** | **11.498** | **<0.0001** | **0.435** |
|  |  | **Moderate** | **42.082** | **5.286** | **<0.0001** | **0.405** |
|  |  | **High** | **38.481** | **8.036** | **<0.0001** | **0.425** |
| 14 | Temporal SD of biomass ~ Shannon H' | **Low** | **43.316** | **8.884** | **<0.0001** | **0.442** |
|  |  | **Moderate** | **34.313** | **4.095** | **<0.0001** | **0.430** |
|  |  | **High** | **29.569** | **6.053** | **<0.0001** | **0.435** |

**Table S4** Results for bivariate correlation analyses based on ordinary least squares linear regression models (LMs) were obtained for the interval between 2009 and 2018, within a 10-year period. These analyses were conducted at three distinct levels of trophic complexity (low: N = 32; moderate: N = 95; high: N = 33). *R^2^* is the explained variance in LMs. Bold face denotes *P* ≤ 0.05.

| NO. | Models | Trophic complexities | Estimate | Std. Error | *P-values* | *R^2^* |
| --- | --- | --- | --- | --- | --- | --- |
| 01 | Community stability ~ Species asynchrony | **Low** | **0.886** | **0.135** | **<0.0001** | **0.588** |
|  |  | **Moderate** | **0.890** | **0.068** | **<0.0001** | **0.647** |
|  |  | **High** | **0.865** | **0.124** | **<0.0001** | **0.612** |
| 02 | Community stability ~ Species stability | **Low** | **0.739** | **0.275** | **0.0115** | **0.195** |
|  |  | **Moderate** | **0.728** | **0.188** | **0.0002** | **0.139** |
|  |  | **High** | **0.849** | **0.289** | **0.0061** | **0.218** |
| 03 | Community stability ~ Temporal mean of biomass | **Low** | **0.003** | **0.001** | **<0.0001** | **0.459** |
|  |  | **Moderate** | **0.003** | **0.0004** | **<0.0001** | **0.440** |
|  |  | **High** | **0.004** | **0.001** | **<0.0001** | **0.540** |
| 04 | Community stability ~ Temporal SD of biomass | Low | 0.002 | 0.002 | 0.1704 | 0.062 |
|  |  | **Moderate** | **0.004** | **0.001** | **0.0017** | **0.101** |
|  |  | **High** | **0.007** | **0.002** | **0.0063** | **0.217** |
| 05 | Community stability ~ ${ENS}_{PIE}$ | **Low** | **0.480** | **0.114** | **0.0002** | **0.371** |
|  |  | **Moderate** | **0.478** | **0.058** | **<0.0001** | **0.421** |
|  |  | **High** | **0.638** | **0.106** | **<0.0001** | **0.541** |
| 06 | Community stability ~ Shannon H' | **Low** | **0.383** | **0.087** | **0.0001** | **0.393** |
|  |  | **Moderate** | **0.388** | **0.045** | **<0.0001** | **0.442** |
|  |  | **High** | **0.500** | **0.080** | **<0.0001** | **0.555** |
| 07 | Species asynchrony ~ ${ENS}_{PIE}$ | **Low** | **0.570** | **0.069** | **<0.0001** | **0.696** |
|  |  | **Moderate** | **0.587** | **0.033** | **<0.0001** | **0.776** |
|  |  | **High** | **0.724** | **0.054** | **<0.0001** | **0.853** |
| 08 | Species asynchrony ~ Shannon H' | **Low** | **0.450** | **0.051** | **<0.0001** | **0.720** |
|  |  | **Moderate** | **0.470** | **0.025** | **<0.0001** | **0.795** |
|  |  | **High** | **0.572** | **0.037** | **<0.0001** | **0.888** |
| 09 | Species stability ~ ${ENS}_{PIE}$ | Low | -0.109 | 0.084 | 0.2014 | 0.054 |
|  |  | **Moderate** | **-0.093** | **0.038** | **0.0164** | **0.060** |
|  |  | High | -0.067 | 0.085 | 0.4371 | 0.020 |
| 10 | Species stability ~ Shannon H' | Low | -0.083 | 0.065 | 0.2082 | 0.052 |
|  |  | **Moderate** | **-0.072** | **0.030** | **0.0178** | **0.059** |
|  |  | High | -0.054 | 0.066 | 0.4128 | 0.022 |
| 11 | Temporal mean of biomass ~ ${ENS}_{PIE}$ | **Low** | **130.538** | **20.532** | **<0.0001** | **0.574** |
|  |  | **Moderate** | **115.724** | **10.483** | **<0.0001** | **0.567** |
|  |  | **High** | **114.504** | **16.748** | **<0.0001** | **0.601** |
| 12 | Temporal mean of biomass ~ Shannon H' | **Low** | **103.772** | **15.406** | **<0.0001** | **0.602** |
|  |  | **Moderate** | **95.389** | **7.817** | **<0.0001** | **0.616** |
|  |  | **High** | **91.361** | **12.340** | **<0.0001** | **0.639** |
| 13 | Temporal SD of biomass ~ ${ENS}_{PIE}$ | **Low** | **48.335** | **12.137** | **0.0004** | **0.346** |
|  |  | **Moderate** | **37.976** | **5.302** | **<0.0001** | **0.356** |
|  |  | **High** | **36.926** | **8.436** | **0.0001** | **0.382** |
| 14 | Temporal SD of biomass ~ Shannon H' | **Low** | **37.556** | **9.417** | **0.0004** | **0.346** |
|  |  | **Moderate** | **30.981** | **4.122** | **<0.0001** | **0.378** |
|  |  | **High** | **28.897** | **6.486** | **0.0001** | **0.390** |

**Table S5** Results for bivariate correlation analyses based on ordinary least squares linear regression models (LMs) were obtained for the interval between 2009 and 2013, within a 5-year period. These analyses were conducted at three distinct levels of trophic complexity (low: N = 32; moderate: N = 95; high: N = 33). *R^2^* is the explained variance in LMs. Bold face denotes *P* ≤ 0.05.

| NO. | Models | Trophic complexities | Estimate | Std. Error | *P-values* | *R^2^* |
| --- | --- | --- | --- | --- | --- | --- |
| 01 | Community stability ~ Species asynchrony | **Low** | **0.928** | **0.183** | **<0.0001** | **0.461** |
|  |  | **Moderate** | **1.089** | **0.095** | **<0.0001** | **0.587** |
|  |  | **High** | **1.136** | **0.134** | **<0.0001** | **0.699** |
| 02 | Community stability ~ Species stability | **Low** | **0.839** | **0.205** | **0.0003** | **0.359** |
|  |  | **Moderate** | **0.894** | **0.167** | **<0.0001** | **0.237** |
|  |  | **High** | **0.880** | **0.383** | **0.0284** | **0.146** |
| 03 | Community stability ~ Temporal mean of biomass | **Low** | **0.003** | **0.001** | **0.0007** | **0.320** |
|  |  | **Moderate** | **0.002** | **0.001** | **<0.0001** | **0.245** |
|  |  | **High** | **0.004** | **0.001** | **0.0119** | **0.187** |
| 04 | Community stability ~ Temporal SD of biomass | Low | -0.0003 | 0.002 | 0.8990 | 0.001 |
|  |  | Moderate | 0.001 | 0.001 | 0.3596 | 0.009 |
|  |  | High | 0.001 | 0.004 | 0.6997 | 0.005 |
| 05 | Community stability ~ ${ENS}_{PIE}$ | Low | 0.304 | 0.161 | 0.0683 | 0.107 |
|  |  | **Moderate** | **0.354** | **0.062** | **<0.0001** | **0.262** |
|  |  | **High** | **0.560** | **0.194** | **0.0071** | **0.212** |
| 06 | Community stability ~ Shannon H' | Low | 0.252 | 0.126 | 0.0555 | 0.117 |
|  |  | **Moderate** | **0.288** | **0.048** | **<0.0001** | **0.275** |
|  |  | **High** | **0.441** | **0.151** | **0.0064** | **0.217** |
| 07 | Species asynchrony ~ ${ENS}_{PIE}$ | **Low** | **0.420** | **0.098** | **0.0002** | **0.379** |
|  |  | **Moderate** | **0.385** | **0.031** | **<0.0001** | **0.625** |
|  |  | **High** | **0.616** | **0.117** | **<0.0001** | **0.473** |
| 08 | Species asynchrony ~ Shannon H' | **Low** | **0.340** | **0.076** | **0.0001** | **0.397** |
|  |  | **Moderate** | **0.310** | **0.024** | **<0.0001** | **0.645** |
|  |  | **High** | **0.488** | **0.089** | **<0.0001** | **0.490** |
| 09 | Species stability ~ ${ENS}_{PIE}$ | Low | -0.144 | 0.119 | 0.2328 | 0.047 |
|  |  | **Moderate** | **-0.086** | **0.038** | **0.0255** | **0.053** |
|  |  | High | -0.109 | 0.093 | 0.2479 | 0.043 |
| 10 | Species stability ~ Shannon H' | Low | -0.112 | 0.094 | 0.2404 | 0.046 |
|  |  | **Moderate** | **-0.068** | **0.030** | **0.0271** | **0.051** |
|  |  | High | -0.087 | 0.072 | 0.2387 | 0.044 |
| 11 | Temporal mean of biomass ~ ${ENS}_{PIE}$ | **Low** | **115.216** | **19.366** | **<0.0001** | **0.541** |
|  |  | **Moderate** | **101.209** | **9.949** | **<0.0001** | **0.527** |
|  |  | **High** | **104.236** | **16.002** | **<0.0001** | **0.578** |
| 12 | Temporal mean of biomass ~ Shannon H' | **Low** | **93.795** | **14.733** | **<0.0001** | **0.575** |
|  |  | **Moderate** | **83.474** | **7.530** | **<0.0001** | **0.569** |
|  |  | **High** | **83.781** | **11.854** | **<0.0001** | **0.617** |
| 13 | Temporal SD of biomass ~ ${ENS}_{PIE}$ | **Low** | **51.240** | **12.942** | **0.0004** | **0.343** |
|  |  | **Moderate** | **37.642** | **5.622** | **<0.0001** | **0.325** |
|  |  | **High** | **34.663** | **8.312** | **0.0002** | **0.359** |
| 14 | Temporal SD of biomass ~ Shannon H' | **Low** | **40.637** | **10.205** | **0.0004** | **0.346** |
|  |  | **Moderate** | **30.729** | **4.396** | **<0.0001** | **0.344** |
|  |  | **High** | **27.379** | **6.409** | **0.0002** | **0.371** |

**Table S6** Standardized and unstandardized direct effects in our final SEMs from Fig. 4, using ${ENS}_{PIE}$ (i.e., the inverse of Simpson index) to represent realized plant diversity, analyzed over 2009−2023 (15 years). Significant effects (*P* ≤ 0.05) are shown in bold text.

| NO. | Associations (Pathways) | Trophic complexities | Estimate | St. Error | *df* | *P-values* | St. Estimate |
| --- | --- | --- | --- | --- | --- | --- | --- |
| 01 | Plant species richness → ${ENS}_{PIE}$ | **Low** | **0.1312** | **0.0119** | **30** | **<0.0001** | **0.8953** |
|  |  | **Moderate** | **0.1481** | **0.0068** | **93** | **<0.0001** | **0.9142** |
|  |  | **High** | **0.137** | **0.009** | **31** | **<0.0001** | **0.9395** |
| 02 | Plant species richness → Species stability | Low | -0.0124 | 0.0298 | 29 | 0.6810 | -0.1645 |
|  |  | Moderate | -0.0007 | 0.0155 | 92 | 0.9629 | -0.0114 |
|  |  | High | -0.0252 | 0.0437 | 30 | 0.5691 | -0.2964 |
| 03 | Plant species richness → Species asynchrony | **Low** | **0.0459** | **0.0169** | **29** | **0.0112** | **0.4898** |
|  |  | **Moderate** | **0.0606** | **0.0092** | **92** | **<0.0001** | **0.5794** |
|  |  | High | -0.0044 | 0.0228 | 30 | 0.8481 | -0.0342 |
| 04 | ${ENS}_{PIE}$ →  Species stability | Low | -0.0797 | 0.2036 | 29 | 0.6984 | -0.1551 |
|  |  | Moderate | -0.1223 | 0.0957 | 92 | 0.2045 | -0.3113 |
|  |  | High | 0.0231 | 0.2997 | 30 | 0.9390 | 0.0398 |
| 05 | ${ENS}_{PIE}$ →  Species asynchrony | **Low** | **0.2787** | **0.1156** | **29** | **0.0225** | **0.4358** |
|  |  | **Moderate** | **0.2454** | **0.0568** | **92** | **<0.0001** | **0.3802** |
|  |  | **High** | **0.8592** | **0.1562** | **30** | **<0.0001** | **0.9752** |
| 06 | Species stability → Community stability | **Low** | **1.0692** | **0.0538** | **29** | **<0.0001** | **0.7771** |
|  |  | **Moderate** | **1.1153** | **0.0355** | **92** | **<0.0001** | **0.6827** |
|  |  | **High** | **1.0729** | **0.056** | **30** | **<0.0001** | **0.6832** |
| 07 | Species asynchrony → Community stability | **Low** | **0.9542** | **0.0432** | **29** | **<0.0001** | **0.8633** |
|  |  | **Moderate** | **0.9353** | **0.0216** | **92** | **<0.0001** | **0.9406** |
|  |  | **High** | **0.8966** | **0.037** | **30** | **<0.0001** | **0.8640** |

**Table S7** Standardized and unstandardized direct effects in our final SEMs, using ${ENS}_{PIE}$ (i.e., the inverse of Simpson index) to represent realized plant diversity, analyzed over 2009−2018 (10 years). Significant effects (*P* ≤ 0.05) are shown in bold text.

| NO. | Associations (Pathways) | Trophic complexities | Estimate | St. Error | *df* | *P-values* | St. Estimate |
| --- | --- | --- | --- | --- | --- | --- | --- |
| 01 | Plant species richness → ${ENS}_{PIE}$ | **Low** | **0.1443** | **0.0126** | **30** | **<0.0001** | **0.9027** |
|  |  | **Moderate** | **0.1640** | **0.0069** | **93** | **<0.0001** | **0.9269** |
|  |  | **High** | **0.1474** | **0.0096** | **31** | **<0.0001** | **0.9400** |
| 02 | Plant species richness → Species stability | Low | -0.0133 | 0.0315 | 29 | 0.6763 | -0.1766 |
|  |  | Moderate | -0.0026 | 0.0179 | 92 | 0.8854 | -0.0389 |
|  |  | High | -0.0436 | 0.0388 | 30 | 0.2706 | -0.5827 |
| 03 | Plant species richness → Species asynchrony | Low | 0.0380 | 0.0250 | 29 | 0.1390 | 0.3482 |
|  |  | **Moderate** | **0.0521** | **0.0145** | **92** | **0.0005** | **0.4420** |
|  |  | High | 0.0291 | 0.0246 | 30 | 0.2469 | 0.2366 |
| 04 | ${ENS}_{PIE}$ →  Species stability | Low | -0.0341 | 0.1969 | 29 | 0.8635 | -0.0726 |
|  |  | Moderate | -0.0789 | 0.1014 | 92 | 0.4384 | -0.2095 |
|  |  | High | 0.1946 | 0.2477 | 30 | 0.4384 | 0.4078 |
| 05 | ${ENS}_{PIE}$ →  Species asynchrony | **Low** | **0.3552** | **0.1564** | **29** | **0.0307** | **0.5201** |
|  |  | **Moderate** | **0.3138** | **0.0821** | **92** | **0.0002** | **0.4709** |
|  |  | **High** | **0.5498** | **0.1570** | **30** | **0.0015** | **0.7014** |
| 06 | Species stability → Community stability | **Low** | **1.0685** | **0.0499** | **29** | **<0.0001** | **0.6373** |
|  |  | **Moderate** | **1.1259** | **0.0406** | **92** | **<0.0001** | **0.5756** |
|  |  | **High** | **1.1024** | **0.0577** | **30** | **<0.0001** | **0.6066** |
| 07 | Species asynchrony → Community stability | **Low** | **1.0451** | **0.0343** | **29** | **<0.0001** | **0.9053** |
|  |  | **Moderate** | **1.0289** | **0.0229** | **92** | **<0.0001** | **0.9300** |
|  |  | **High** | **0.9716** | **0.0351** | **30** | **<0.0001** | **0.8784** |

**Table S8** Standardized and unstandardized direct effects in our final SEMs, using ${ENS}_{PIE}$ (i.e., the inverse of Simpson index) to represent realized plant diversity, analyzed over 2009−2013 (5 years). Significant effects (*P* ≤ 0.05) are shown in bold text.

| NO. | Associations (Pathways) | Trophic complexities | Estimate | St. Error | *df* | *P-values* | St. Estimate |
| --- | --- | --- | --- | --- | --- | --- | --- |
| 01 | Plant species richness → ${ENS}_{PIE}$ | **Low** | **0.1533** | **0.0128** | **30** | **<0.0001** | **0.9089** |
|  |  | **Moderate** | **0.1805** | **0.0077** | **93** | **<0.0001** | **0.9247** |
|  |  | **High** | **0.1550** | **0.0103** | **31** | **<0.0001** | **0.9380** |
| 02 | Plant species richness → Species stability | Low | -0.0215 | 0.0486 | 29 | 0.6612 | -0.1919 |
|  |  | Moderate | -0.0082 | 0.0195 | 92 | 0.6741 | -0.1123 |
|  |  | High | -0.0011 | 0.0449 | 30 | 0.9807 | -0.0126 |
| 03 | Plant species richness → Species asynchrony | Low | 0.0763 | 0.0378 | 29 | 0.0529 | 0.6630 |
|  |  | **Moderate** | **0.0450** | **0.0152** | **92** | **0.0039** | **0.4744** |
|  |  | High | -0.0257 | 0.0564 | 30 | 0.6522 | -0.1734 |
| 04 | ${ENS}_{PIE}$ →  Species stability | Low | -0.0284 | 0.2882 | 29 | 0.9223 | -0.0426 |
|  |  | Moderate | -0.0471 | 0.1002 | 92 | 0.6391 | -0.1253 |
|  |  | High | -0.103 | 0.2719 | 30 | 0.7075 | -0.1952 |
| 05 | ${ENS}_{PIE}$ →  Species asynchrony | Low | 0.0092 | 0.2242 | 29 | 0.9677 | 0.0134 |
|  |  | **Moderate** | **0.1711** | **0.0780** | **92** | **0.0307** | **0.3518** |
|  |  | **High** | **0.7622** | **0.3412** | **30** | **0.0331** | **0.8507** |
| 06 | Species stability → Community stability | **Low** | **1.0932** | **0.0351** | **29** | **<0.0001** | **0.8001** |
|  |  | **Moderate** | **1.2279** | **0.0282** | **92** | **<0.0001** | **0.8640** |
|  |  | **High** | **1.2176** | **0.0628** | **30** | **<0.0001** | **0.8962** |
| 07 | Species asynchrony → Community stability | **Low** | **1.0248** | **0.0360** | **29** | **<0.0001** | **0.7313** |
|  |  | **Moderate** | **1.1430** | **0.0364** | **92** | **<0.0001** | **0.6219** |
|  |  | **High** | **1.1336** | **0.1066** | **30** | **<0.0001** | **0.4915** |

**Table S9** Standardized and unstandardized direct effects in our final SEMs from Fig. S7, using the Shannon H' index to represent realized plant diversity, analyzed over 2009−2023 (15 years). Significant effects (*P* ≤ 0.05) are shown in bold text.

| NO. | Associations (Pathways) | Trophic complexities | Estimate | St. Error | *df* | *P-values* | St. Estimate |
| --- | --- | --- | --- | --- | --- | --- | --- |
| 01 | Plant species richness → Shannon H' | **Low** | **0.1728** | **0.0137** | **30** | **<0.0001** | **0.9171** |
|  |  | **Moderate** | **0.1906** | **0.0077** | **93** | **<0.0001** | **0.9314** |
|  |  | **High** | **0.1833** | **0.0102** | **31** | **<0.0001** | **0.9551** |
| 02 | Plant species richness → Species stability | Low | -0.0137 | 0.0334 | 29 | 0.6848 | -0.1817 |
|  |  | Moderate | 0.0005 | 0.0173 | 92 | 0.9756 | 0.0083 |
|  |  | High | -0.0298 | 0.0505 | 30 | 0.5600 | -0.3506 |
| 03 | Plant species richness → Species asynchrony | **Low** | **0.0391** | **0.0188** | **29** | **0.0464** | **0.4170** |
|  |  | **Moderate** | **0.0553** | **0.0102** | **92** | **<0.0001** | **0.5286** |
|  |  | High | -0.0431 | 0.0223 | 30 | 0.0629 | -0.3354 |
| 04 | Shannon H' →  Species stability | Low | -0.0530 | 0.1771 | 29 | 0.7668 | -0.1327 |
|  |  | Moderate | -0.1016 | 0.0844 | 92 | 0.2318 | -0.3267 |
|  |  | High | 0.0424 | 0.2630 | 30 | 0.8730 | 0.0959 |
| 05 | Shannon H' →  Species asynchrony | **Low** | **0.2511** | **0.0997** | **29** | **0.0175** | **0.5049** |
|  |  | **Moderate** | **0.2185** | **0.0499** | **92** | **<0.0001** | **0.4277** |
|  |  | **High** | **0.8531** | **0.1162** | **30** | **<0.0001** | **1.2746** |
| 06 | Species stability → Community stability | **Low** | **1.0692** | **0.0538** | **29** | **<0.0001** | **0.7771** |
|  |  | **Moderate** | **1.1153** | **0.0355** | **92** | **<0.0001** | **0.6827** |
|  |  | **High** | **1.0729** | **0.0560** | **30** | **<0.0001** | **0.6832** |
| 07 | Species asynchrony → Community stability | **Low** | **0.9542** | **0.0432** | **29** | **<0.0001** | **0.8633** |
|  |  | **Moderate** | **0.9353** | **0.0216** | **92** | **<0.0001** | **0.9406** |
|  |  | **High** | **0.8966** | **0.0370** | **30** | **<0.0001** | **0.8640** |

**Table S10** Standardized and unstandardized direct effects in our final SEMs, using the Shannon H' index to represent realized plant diversity, analyzed over 2009−2018 (10 years). Significant effects (*P* ≤ 0.05) are shown in bold text.

| NO. | Associations (Pathways) | Trophic complexities | Estimate | St. Error | *df* | *P-values* | St. Estimate |
| --- | --- | --- | --- | --- | --- | --- | --- |
| 01 | Plant species richness → Shannon H' | **Low** | **0.1892** | **0.0149** | **30** | **<0.0001** | **0.9186** |
|  |  | **Moderate** | **0.2109** | **0.0077** | **93** | **<0.0001** | **0.9430** |
|  |  | **High** | **0.1933** | **0.0108** | **31** | **<0.0001** | **0.9547** |
| 02 | Plant species richness → Species stability | Low | -0.0155 | 0.0343 | 29 | 0.6554 | -0.2056 |
|  |  | Moderate | -0.0026 | 0.0203 | 92 | 0.8981 | -0.0390 |
|  |  | High | -0.0495 | 0.0445 | 30 | 0.2745 | -0.6619 |
| 03 | Plant species richness → Species asynchrony | Low | 0.0269 | 0.0267 | 29 | 0.3218 | 0.2466 |
|  |  | **Moderate** | **0.0400** | **0.0162** | **92** | **0.0154** | **0.3390** |
|  |  | High | -0.0048 | 0.0253 | 30 | 0.8498 | -0.0393 |
| 04 | Shannon H' →  Species stability | Low | -0.0145 | 0.1664 | 29 | 0.9311 | -0.0398 |
|  |  | Moderate | -0.0613 | 0.0906 | 92 | 0.5001 | -0.2058 |
|  |  | High | 0.1789 | 0.2196 | 30 | 0.4216 | 0.4844 |
| 05 | Shannon H' →  Species asynchrony | **Low** | **0.3297** | **0.1297** | **29** | **0.0166** | **0.6218** |
|  |  | **Moderate** | **0.3016** | **0.0724** | **92** | **0.0001** | **0.5721** |
|  |  | **High** | **0.5944** | **0.1248** | **30** | **<0.0001** | **0.9796** |
| 06 | Species stability → Community stability | **Low** | **1.0685** | **0.0499** | **29** | **<0.0001** | **0.6373** |
|  |  | **Moderate** | **1.1259** | **0.0406** | **92** | **<0.0001** | **0.5756** |
|  |  | **High** | **1.1024** | **0.0577** | **30** | **<0.0001** | **0.6066** |
| 07 | Species asynchrony → Community stability | **Low** | **1.0451** | **0.0343** | **29** | **<0.0001** | **0.9053** |
|  |  | **Moderate** | **1.0289** | **0.0229** | **92** | **<0.0001** | **0.9300** |
|  |  | **High** | **0.9716** | **0.0351** | **30** | **<0.0001** | **0.8784** |

**Table S11** Standardized and unstandardized direct effects in our final SEMs, using the Shannon H' index to represent realized plant diversity, analyzed over 2009−2013 (5 years). Significant effects (*P* ≤ 0.05) are shown in bold text.

| NO. | Associations (Pathways) | Trophic complexities | Estimate | St. Error | *df* | *P-values* | St. Estimate |
| --- | --- | --- | --- | --- | --- | --- | --- |
| 01 | Plant species richness → Shannon H' | **Low** | **0.1979** | **0.0146** | **30** | **<0.0001** | **0.9271** |
|  |  | **Moderate** | **0.2317** | **0.0086** | **93** | **<0.0001** | **0.9421** |
|  |  | **High** | **0.2036** | **0.0109** | **31** | **<0.0001** | **0.9582** |
| 02 | Plant species richness → Species stability | Low | -0.0260 | 0.0541 | 29 | 0.6345 | -0.2316 |
|  |  | Moderate | -0.0095 | 0.0222 | 92 | 0.6695 | -0.1295 |
|  |  | High | 0.0069 | 0.0544 | 30 | 0.8996 | 0.0793 |
| 03 | Plant species richness → Species asynchrony | Low | 0.0745 | 0.0420 | 29 | 0.0869 | 0.6473 |
|  |  | **Moderate** | **0.0364** | **0.0172** | **92** | **0.0369** | **0.3829** |
|  |  | High | -0.0833 | 0.0657 | 30 | 0.2148 | -0.5625 |
| 04 | Shannon H' →  Species stability | Low | 0.0005 | 0.2533 | 29 | 0.9983 | 0.0010 |
|  |  | Moderate | -0.0313 | 0.0903 | 92 | 0.7299 | -0.1048 |
|  |  | High | -0.1178 | 0.2559 | 30 | 0.6486 | -0.2869 |
| 05 | Shannon H' →  Species asynchrony | Low | 0.0162 | 0.1970 | 29 | 0.9351 | 0.0300 |
|  |  | **Moderate** | **0.1707** | **0.0698** | **92** | **0.0163** | **0.4424** |
|  |  | **High** | **0.8634** | **0.3093** | **30** | **0.0090** | **1.2389** |
| 06 | Species stability → Community stability | **Low** | **1.0248** | **0.0360** | **29** | **<0.0001** | **0.7313** |
|  |  | **Moderate** | **1.1430** | **0.0364** | **92** | **<0.0001** | **0.6219** |
|  |  | **High** | **1.1336** | **0.1066** | **30** | **<0.0001** | **0.4915** |
| 07 | Species asynchrony → Community stability | **Low** | **1.0932** | **0.0351** | **29** | **<0.0001** | **0.8001** |
|  |  | **Moderate** | **1.2279** | **0.0282** | **92** | **<0.0001** | **0.8640** |
|  |  | **High** | **1.2176** | **0.0628** | **30** | **<0.0001** | **0.8962** |

**Table S12** Individual R-squared of response variables in our final SEMs from Fig. 4 and Tables S6−S8, with “*plotID*” as random effects. Note that ${ENS}_{PIE}$ (i.e., the inverse of Simpson index) is used to represent realized plant diversity in these SEMs. The marginal (*R^2^_m_*) and conditional (*R^2^_c_*) r-squared represent “*fixed effects*” and “*fixed + random effects*” explanations, respectively.

| Figures & Tables | Response variables | Low trophic complexity | | Moderate trophic complexity | | High trophic complexity | |
| --- | --- | --- | --- | --- | --- | --- | --- |
|  |  | *R^2^_m_* | *R^2^_c_* | *R^2^_m_* | *R^2^_c_* | *R^2^_m_* | *R^2^_c_* |
| 15-year interval (2009−2023) | | | | | | | |
| Fig. 4 & Table S6 | ${ENS}_{PIE}$ | 0.80 | 0.97 | 0.83 | 0.99 | 0.88 | 0.99 |
|  | Species stability | 0.09 | 0.89 | 0.10 | 0.94 | 0.06 | 0.88 |
|  | Species asynchrony | 0.80 | 0.98 | 0.88 | 0.99 | 0.88 | 0.99 |
|  | Community stability | 0.96 | NA | 0.96 | NA | 0.96 | NA |
| 10-year interval (2009−2018) | | | | | | | |
| Table S7 | ${ENS}_{PIE}$ | 0.88 | 0.99 | 0.86 | 0.99 | 0.88 | 0.99 |
|  | Species stability | 0.06 | 0.88 | 0.06 | 0.94 | 0.06 | 0.88 |
|  | Species asynchrony | 0.88 | 0.99 | 0.80 | 0.99 | 0.85 | 0.98 |
|  | Community stability | 0.96 | NA | 0.96 | NA | 0.97 | NA |
| 5-year interval (2009−2013) | | | | | | | |
| Table S8 | ${ENS}_{PIE}$ | 0.82 | 0.98 | 0.85 | 0.99 | 0.88 | 0.98 |
|  | Species stability | 0.05 | 0.88 | 0.05 | 0.94 | 0.04 | 0.88 |
|  | Species asynchrony | 0.44 | 0.93 | 0.65 | 0.98 | 0.46 | 0.93 |
|  | Community stability | 0.98 | NA | 0.96 | NA | 0.94 | NA |

**Table S13** Individual R-squared of response variables in our final SEMs from Fig. S7 and Tables S9−S11, with “*plotID*” as random effects. Note that the Shannon H' index is used to represent realized plant diversity in these SEMs. The marginal (*R^2^_m_*) and conditional (*R^2^_c_*) r-squared represent “*fixed effects*” and “*fixed + random effects*” explanations, respectively.

| Figures & Tables | Response variables | Low trophic complexity | | Moderate trophic complexity | | High trophic complexity | |  |
| --- | --- | --- | --- | --- | --- | --- | --- | --- |
|  |  | *R^2^_m_* | *R^2^_c_* | *R^2^_m_* | *R^2^_c_* | *R^2^_m_* | *R^2^_c_* |  |
| 15-year interval (2009−2023) | | | | | | | | |
| Fig. S7 & Table S9 | Shannon H' | 0.84 | 0.98 | 0.87 | 0.99 | 0.91 | 0.99 |  |
|  | Species stability | 0.09 | 0.89 | 0.10 | 0.94 | 0.06 | 0.88 |  |
|  | Species asynchrony | 0.80 | 0.98 | 0.88 | 0.99 | 0.92 | 0.99 |  |
|  | Community stability | 0.96 | NA | 0.96 | NA | 0.96 | NA |  |
| 10-year interval (2009−2018) | | | | | | | | |
| Table S10 | Shannon H' | 0.84 | 0.98 | 0.89 | 0.99 | 0.91 | 0.99 |  |
|  | Species stability | 0.06 | 0.88 | 0.06 | 0.94 | 0.06 | 0.88 |  |
|  | Species asynchrony | 0.72 | 0.96 | 0.80 | 0.99 | 0.88 | 0.99 |  |
|  | Community stability | 0.98 | NA | 0.96 | NA | 0.97 | NA |  |
| 5-year interval (2009−2013) | | | | | | | | |
| Table S11 | Shannon H' | 0.86 | 0.98 | 0.89 | 0.99 | 0.92 | 0.99 |  |
|  | Species stability | 0.05 | 0.88 | 0.05 | 0.94 | 0.04 | 0.88 |  |
|  | Species asynchrony | 0.44 | 0.93 | 0.66 | 0.98 | 0.50 | 0.94 |  |
|  | Community stability | 0.98 | NA | 0.96 | NA | 0.94 | NA |  |

**Table S14** Pairwise P-values for differences in a single path between two trophic complexity categories (low, moderate, high) from multigroup SEMs, using the ${ENS}_{PIE}$ (i.e., the inverse of Simpson index) for realized plant diversity. The SEMs were fully unconstrained and run in ‘*lavaan*’ and P-values were calculated by comparing these models to a version where a single path between two of the three trophic complexity categories was constrained (forced to be identical). A significant p-value indicates that the path is statistically significantly different in the two trophic complexity categories. Significant differences (*P* ≤ 0.05) are shown in bold text.

| Pathways | Intervals | Low vs. Moderate | Moderate vs. High | Low vs. High |
| --- | --- | --- | --- | --- |
| Plant species richness → ${ENS}_{PIE}$ | 15-yr | 0.2092 | 0.3158 | 0.6869 |
|  | 10-yr | 0.1619 | 0.1518 | 0.8436 |
|  | 5-yr | 0.0662 | **0.0455** | 0.9135 |
| Plant species richness → Species stability | 15-yr | 0.7177 | 0.5826 | 0.8001 |
|  | 10-yr | 0.7588 | 0.3202 | 0.5253 |
|  | 5-yr | 0.7911 | 0.8790 | 0.7460 |
| Plant species richness → Species asynchrony | 15-yr | 0.4287 | **0.0080** | 0.0667 |
|  | 10-yr | 0.6124 | 0.4033 | 0.7888 |
|  | 5-yr | 0.4243 | 0.2098 | 0.1189 |
| ${ENS}_{PIE}$ → Species stability | 15-yr | 0.8431 | 0.6293 | 0.7660 |
|  | 10-yr | 0.8329 | 0.2892 | 0.4492 |
|  | 5-yr | 0.9487 | 0.8404 | 0.8433 |
| ${ENS}_{PIE}$ → Species asynchrony | 15-yr | 0.7877 | **0.0004** | **0.0026** |
|  | 10-yr | 0.8068 | 0.1691 | 0.3582 |
|  | 5-yr | 0.4765 | 0.0832 | 0.0569 |
| Species stability → Community stability | 15-yr | 0.4586 | 0.5082 | 0.9598 |
|  | 10-yr | 0.3555 | 0.7289 | 0.6410 |
|  | 5-yr | **0.0188** | 0.9305 | 0.3136 |
| Species asynchrony → Community stability | 15-yr | 0.6837 | 0.3489 | 0.2899 |
|  | 10-yr | 0.6830 | 0.1590 | 0.1195 |
|  | 5-yr | **0.0026** | 0.8761 | 0.0741 |

**Table S15** Pairwise P-values for differences in a single path between two trophic complexity categories (low, moderate, high) from multigroup SEMs, using the Shannon H' index for realized plant diversity. The SEMs were fully unconstrained and run in ‘*lavaan*’ and P-values were calculated by comparing these models to a version where a single path between two of the three trophic complexity categories was constrained (forced to be identical). P-values from comparing a fully unconstrained multigroup SEM ran in ‘*lavaan*’ to the same model where one single path between two of the three trophic complexity categories (low, moderate, high) was constrained (forced to be identical). A significant p-value indicates that the path is statistically significantly different in the two trophic complexity categories. Significant differences (*P* ≤ 0.05) are shown in bold text.

| Pathways | Intervals | Low vs. Moderate | Moderate vs. High | Low vs. High |
| --- | --- | --- | --- | --- |
| Plant species richness → Shannon H' | 15-yr | 0.2476 | 0.5615 | 0.5248 |
|  | 10-yr | 0.1866 | 0.1784 | 0.8171 |
|  | 5-yr | **0.0437** | **0.0403** | 0.7473 |
| Plant species richness → Species stability | 15-yr | 0.6935 | 0.5538 | 0.7804 |
|  | 10-yr | 0.7369 | 0.3193 | 0.5253 |
|  | 5-yr | 0.7684 | 0.7704 | 0.6527 |
| Plant species richness → Species asynchrony | 15-yr | 0.4310 | **0.0001** | **0.0042** |
|  | 10-yr | 0.6642 | 0.1244 | 0.3662 |
|  | 5-yr | 0.3820 | 0.0713 | **0.0375** |
| Shannon H' → Species stability | 15-yr | 0.7960 | 0.5864 | 0.7523 |
|  | 10-yr | 0.7968 | 0.2940 | 0.4623 |
|  | 5-yr | 0.9015 | 0.7391 | 0.7303 |
| Shannon H' → Species asynchrony | 15-yr | 0.7604 | **<0.0001** | **0.0001** |
|  | 10-yr | 0.8440 | **0.0382** | 0.1259 |
|  | 5-yr | 0.4406 | **0.0270** | **0.0181** |
| Species stability → Community stability | 15-yr | 0.4586 | 0.5082 | 0.9598 |
|  | 10-yr | 0.3555 | 0.7289 | 0.6410 |
|  | 5-yr | **0.0188** | 0.9305 | 0.3136 |
| Species asynchrony → Community stability | 15-yr | 0.6837 | 0.3489 | 0.2899 |
|  | 10-yr | 0.6830 | 0.1590 | 0.1195 |
|  | 5-yr | **0.0026** | 0.8761 | 0.0741 |
